# Changing with the times: Seasonal environmental gradients unveil dynamic bat assemblages and vulnerability

**DOI:** 10.1002/ece3.10246

**Published:** 2023-07-17

**Authors:** Helena Raposeira, Pedro Horta, Ruben Heleno, Hugo Rebelo

**Affiliations:** ^1^ CIBIO, Research Centre in Biodiversity and Genetic Resources, InBIO Associated Laboratory University of Porto Vairão Portugal; ^2^ Department of Biology, Faculty of Sciences University of Porto Porto Portugal; ^3^ OII – Observatory Inovation Research Linhares Portugal; ^4^ Department of Life Sciences, TERRA Associate Laboratory, Center for Functional Ecology University of Coimbra Coimbra Portugal; ^5^ BIOPOLIS Program in Genomics, Biodiversity and Land Planning CIBIO Vairão Portugal; ^6^ ESS, Instituto Politécnico de Setúbal Setúbal Portugal

**Keywords:** bat assemblages, climate change, ecological plasticity, mountains, seasonality, vulnerability assessment

## Abstract

Uncovering the temporal and spatial dynamics of biological communities in response to biotic and abiotic drivers is essential to predict the effects of environmental change on biodiversity. Similarly, estimating species vulnerability in the face of such dynamics is crucial for implementing effective conservation actions. We explored how bat diversity changes over the year across an altitudinal gradient and identified the environmental drivers that shape bat communities. By analysing species' marginality within the biophysical niche space, we evaluated bats' vulnerability to foreseeable environmental changes. Our results suggest that altitude, the proportion of forest cover and shrub cover are the main drivers shaping bat communities year‐round. Additionally, while some bat species are restricted to a single ecological assemblage (or ecological preferences group), others show greater plasticity throughout the year. Importantly, we found that although bats associated with highland habitats and forests could be particularly vulnerable to environmental changes (in particular *Myotis mystacinus*), this vulnerability correlates poorly with their national conservation status. We suggest that species' ecological plasticity is critical for the resilience of biological communities exposed to environmental changes and should be considered when planning tailored conservation strategies.

## Graphical abstract

We assessed bat vulnerability by their seasonal needs across altitudinal gradients. The altitudinal gradient, tree and shrub cover drove the bat community. Bats associated with highland habitats and forests are mainly vulnerable to climate change. Bats' ecological plasticity is critical for the resilience of communities under climate change. Studying vulnerability with ecological plasticity improves species risk assessment.

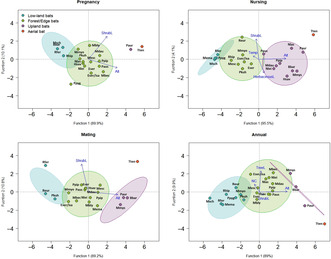

## 1 INTRODUCTION

Understanding why some species tend to co‐occur in space and time is a central goal of community ecology (Morin, 2009). In turn, understanding how species interact with other co‐occurring species to form dynamic ecological communities is important to estimate their potential to deal with environmental changes (Lurgi et al., 2012; Razgour et al., 2019).

Bats are one of the most diverse orders of mammals, with many of those species forming complex subsets due to their similar ecological requirements. Here, we define ‘assemblages' as a subset of a bat community defined by taxonomic reasons (Patterson et al., 2003). However, these subsets are likely affected by multiple factors, whose relative importance is still poorly understood, hindering our ability to predict how species may interact (Patterson et al., 2003).On the contrary, bat species will be affected by different global change drivers (Jones et al., 2009; Koivula et al., 2018; Walther, 2010). These drivers include, for example, seasonal cycles (Parmesan, 2006) or/and extreme events such as storms, hurricanes, severe droughts or wildfires (Ancillotto et al., 2021; Jones et al., 2009), which are increasing in frequency due to anthropogenic global changes (Hooper et al., 2005; Jones et al., 2009). All these factors have been reported to affect the composition and functioning of the subsets of bat communities (Blakey et al., 2019; Craig et al., 1994; Oliveira et al., 2022; Pruvot et al., 2019; Walther, 2010).

The composition of bat assemblages depends primarily on each species' ecological requirements, and on their interspecific interactions (Morin, 2009). In particular, species with high plasticity and generalist ecological requirements will tend to blur the boundaries of species assemblages, while species with narrow and stringent requirements will lead to a stronger differentiation between species' assemblages (Korñan & Kropil, 2014). Bats are an excellent group to explore this topic because they present a highly variable ecological plasticity, high dispersal capacity (Oelbaum et al., 2019) and a complex biological cycle (Dietz et al., 2009) and can also show strong overlap in ecological requirements (Oelbaum et al., 2019).

Bat ecologists frequently study subsets of ecological communities due to logistics constraints, developing, by definition, ‘the ecological study of chiropteran assemblages’ (Patterson et al., 2003). Other researchers defined several types of guilds on bat communities, taking into account a few functional characteristics, such as habitat use, echolocation characteristics (Denzinger & Schnitzler, 2013), or diet composition (Oelbaum et al., 2019). However, recent analyses of bats’ diet composition, foraging strategies and morphological traits suggest that those traditional bat trophic guilds tend to significantly overlap (Gordon et al., 2019; Oelbaum et al., 2019). Therefore, if these species groups are based only on one or a few static variables, they can lead to misleading or artificial classifications.

Ecological communities are intrinsically dynamic, with species occurrence and ecological relationships changing over time due to species‐specific responses to seasonal environmental changes (Kalyuzhny et al., 2019). For example, the percentage of canopy cover changes seasonally throughout the year with the leaf burst and senescence, influencing the presence and abundance of insect prey and refuge for bats (Wehr et al., 2016). Bailey et al. (2019) showed that canopy cover tends to promote bat species richness and abundance. Yet, few studies have explored the seasonal impact of environmental conditions on bat communities across environmental gradients, particularly their implication on bat assemblages and species composition (Adams & Thibault, 2006; Beilke et al., 2021; Castro et al., 2019).

This is relevant because the adaptation capacity of species over time is likely related to their level of susceptibility to environmental changes. This information on each species requirements and vulnerabilities throughout the year is likely critical to inform conservation managers and decision‐makers.

With a high level of biological richness (one‐third of the global terrestrial biodiversity) (Spehn et al., 2011), mountains offer excellent opportunities to evaluate how environmental gradients shape community composition over time. The marked altitudinal variation of climatic conditions gives rise to a range of vegetation types (Körner, 2004), which together with topographic features results in pronounced seasonality of the climatic conditions and the biophysical environment. On the contrary, the steep climatic and altitudinal gradients also provide valuable opportunities to predict species' long‐term ecological responses to climate warming (Mayor et al., 2017) and to explore the drivers of community composition (Jansen et al., 2002; Sillero et al., 2009). Species vulnerability is a key concept in conservation biology. Vulnerability reflects the proximity of subjects (e.g. populations, species and communities) to destructive or disturbing factors (Pressey et al., 1996). Vulnerability assessments have been extensively used to inform the management of terrestrial and marine resources and communities on either global or regional scales according to different management objectives (Comte & Olden, 2017; Morrison et al., 2016; Morzaria‐Luna et al., 2014; Welle & Birkmann, 2015). There are several possible approaches to assess species vulnerability (Pacifici et al., 2015) such as detecting sharp declines in species population size and/or distribution range (Huntley et al., 2012; Razgour et al., 2019; Sattler et al., 2007; IUCN & Petitions Subcommittee, 2022). Yet, this information is seldom available for a vast array of *taxa*. Alternatively, vulnerability assessments using biophysical gradients can anticipate population threats and provide a quantitative assessment that can be useful to guide conservation efforts and fine‐tune conservation policy and practice (Pressey & Taffs, 2001; Shokri & Gladstone, 2013). However, estimating species and community vulnerability is far from trivial (Tanalgo et al., 2018) and particularly predicting how seasonal variability affects species vulnerability to environmental changes (Meyer et al., 2008; Welman et al., 2017; Zamora‐Gutierrez et al., 2021). Therefore, species' niche marginality can be a valuable indicator of their vulnerability to environmental changes (Sattler et al., 2007) as it reflects species persistence probability under future environmental changes (Shreeve et al., 1996). It is included in the ecological‐niche factor analysis (ENFA) approach, and it has been widely used to model species' distribution (Sattler et al., 2007) but also for wildlife management, habitat assessment and habitat prediction (Hirzel et al., 2002; Ouyang & Liu, 2008). Recently, Rinnan and Lawler (2019) adapted the ENFA method to quantify species' vulnerability to climate change using spatial data and future projections of global climate models. Marginality appears to be a useful tool to assess vulnerability in conjugation with other analyses (Rinnan & Lawler, 2019).

The main aim of this research was to identify the key environmental drivers of bats' distribution along a strong and highly dynamic biophysical gradient associated with a mountain range, taking into account seasonality. We then estimated species marginality to environmental conditions and provide a novel vulnerability assessment protocol that incorporates species requirements and the direction of foreseeable environmental changes. Specifically, we address three main questions: (1) What environmental variables shape bat communities throughout the year? (2) Which bat species are associated with which ecological assemblages in response to seasonality? (3) Which bat species are more vulnerable to climate change, given their environmental requirements and is this vulnerability reflected in their conservation status?

## 2 METHODS

### 2.1 Study area

**FIGURE 1:**
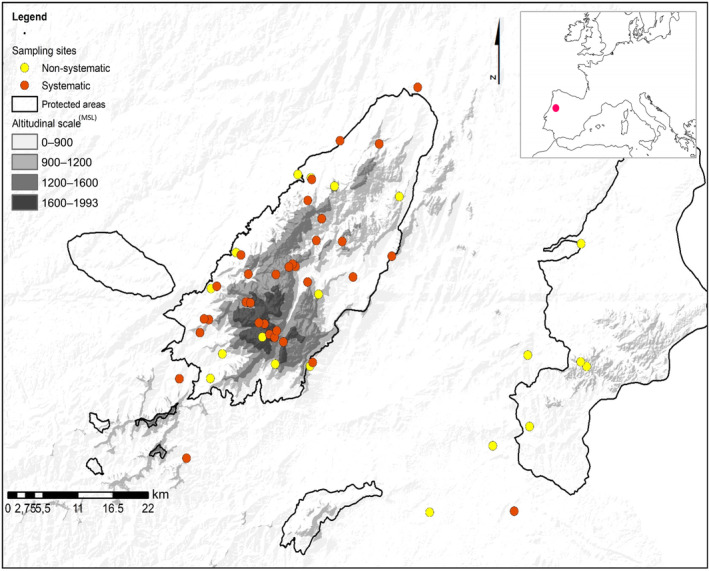
Altitudinal map and location of the study area, Serra da Estrela mountain range—Portugal, including the location of the systematic and nonsystematic sampling sites. The coordinates of sampling sites are available in the Data S1.

The sampling area, located at the mountain chain of Serra da Estrela (central Portugal), was selected considering the environmental gradient shaped by two major bioclimatic influences: Temperate (colder and humid) northern influence and Mediterranean (hotter and dryer, with more pronounced seasons) south‐eastern influence (Figure 1) (Jansen & Correia, 2002). Serra da Estrela (maximum altitude 1993 m MSL) has a wide range of different habitats in a relatively small area, wherein ecological assessments are still scarce (Figure S9).

### 2.2 Approach

**FIGURE 2:**
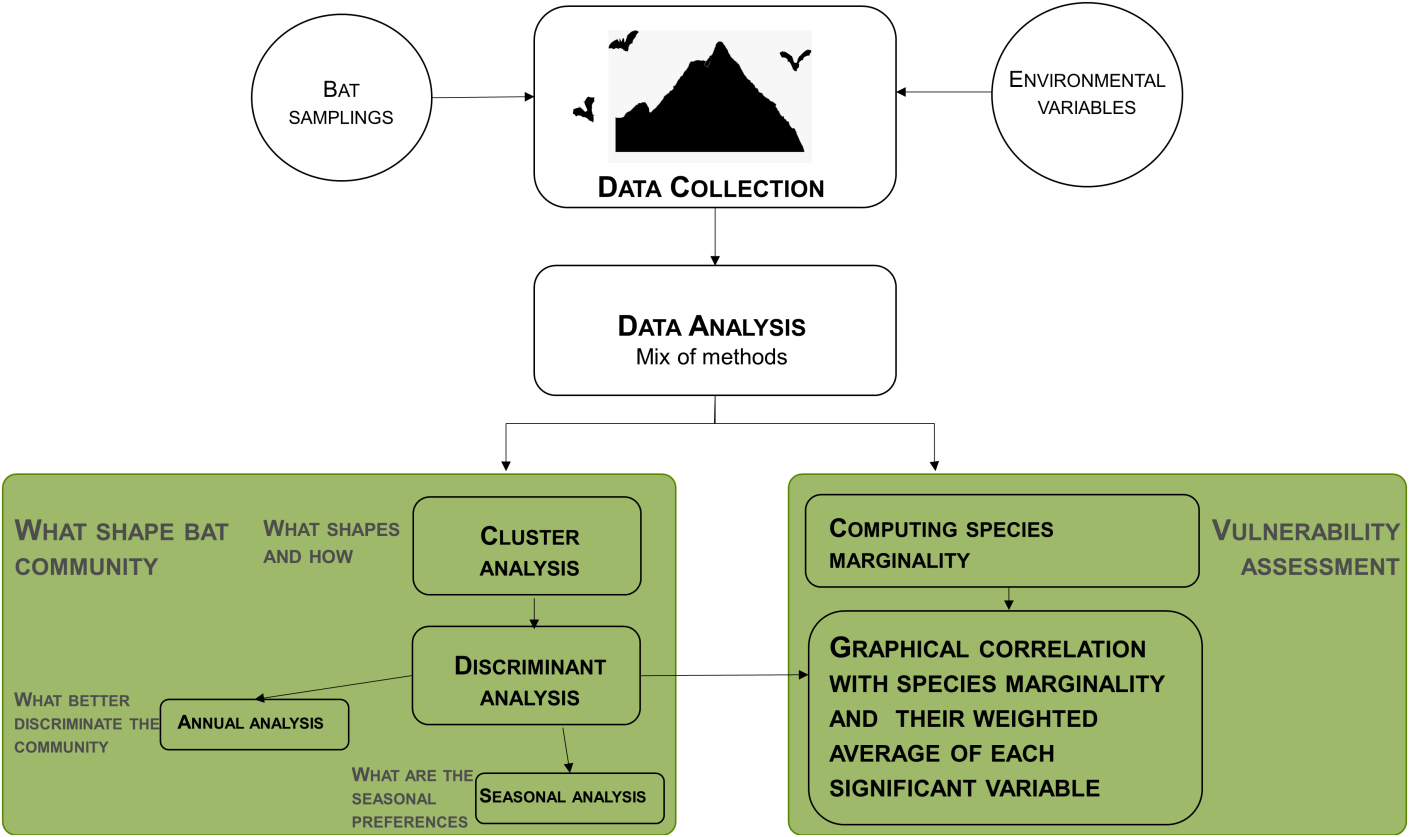
Approach scheme developed for survey data and analysis.

To answer our research questions, we sampled bats at 53 sites located according to a stratified randomization, where 34 sites were visited periodically (at least one visit per season per sampling site; Figure 2; Table S23). The remaining sample sites were located in areas aiming to cover the rarest species and habitats of the study area. We then applied two different statistical methods to understand how the environment shapes the composition of bat communities. First, we applied a cluster analysis to group bat species according to their co‐occurrences (i.e. assemblages). We then used a discriminant analysis to identify the most relevant environmental variables associated with the ecological preferences of each assemblage (Figure 1). We repeated this analysis for each season to identify the specific preferences covering each part of bats' reproductive cycle and for the entire year. These two levels of analysis should thus be regarded as complementary. Lastly, we computed for each species the marginality for each significant variable and correlated it, graphically, with respective weighted average to quantify the vulnerability of each species and identify the most discriminating environmental features of each ecological assemblage (Figure 1).

### 2.3 Sampling design

Between May 2017 and October 2018, 34 sites (Figure 1) were sampled at least once on each of the three biological active seasons (Mean ± SD = 3.16 ± 0.45 sampling visits per site) of the bat's biological cycle, namely: pregnancy (May/June), nursing (July/August) and mating (September/October; Lourenço & Palmeirim, 2008). The sampling sites were selected in order to cover the entire altitudinal and environmental gradients proportionally to their availability in the study area (Figure 1). At each site, bats were captured with mist nets placed always at the same locations. Additionally, 19 sites were sampled nonsystematically (Figure 1) to capture underrepresented conditions and increase dataset representability. Bats were captured in free‐flight with mist nets (two‐ply, 3.81 cm mesh; Avinet, Inc.) at water points and foraging sites. Depending on the type of sampling site, we used triple high, double and/or simple nets that always totalized 369.2 m^2^ of nets' area per sampling site. The length of each individual net varied between 18 m and 6 m with a height of 8 to 2.6 m. Nets were opened at least during the first 5 h of activity, starting 30 min after sunset. However, the actual period of each survey was limited by the weather conditions. Thus, relative abundances are presented as number of captures/hour following Gannon and Willig (1998). Bats were identified based on a morphological identification guide (Dietz & von Helversen, 2004). The two cryptic species *Eptesicus serotinus* and *E. isabellinus* (Ibáñez et al., 2006; Santos et al., 2014) were distinguished whenever possible based on dentition measurement (CM3 > 8.4 mm = *E. serotinus*; CM3 < 8.4 mm = *E. serotinus/isabellinus* complex) according to Horta et al. (2022). When this was not possible, these individuals were registered as a sister species complex in which the occurrence of hybrids is possible (Centeno‐Cuadros et al., 2019; Horta et al., 2022).

During bat trapping sessions, we measured the weather conditions during sampling through a pocket weather meter—Kestrel 3000 and by direct observation (namely wind speed, temperature, humidity, cloudiness, visibility, pluviosity and type of pluviosity). The altitude was measured by GPS, and the night cooling metric resulted from the difference between the temperature at the beginning and end of the night (Table 1). The landscape was characterized by determining each landscape variable percentage cover within a 200 m radius, namely tree layer, shrub layer, herbaceous layer and vegetation cover. The classification of the vegetation categories was based on the average vegetation height within the radius (Table 1). The vegetation structure is a qualitative variable which was assessed by direct observation during sampling based on five categories, similar to as proposed by Martí and del Moral (2003): herbaceous (grasslands, prairies, swards, etc.), brushwood (with a great diversity of nonwoody medium shrubs), open forest, young forest (dense forest with tree layer height <12 m) and mature dense forest (tree layer >12 m; Table 1). All biophysical variables were collected throughout the bats' activity biological cycle (Wehr et al., 2016).

**TABLE 1:** Environmental variables collected in each sampling site.

Variables	Acronyms	Units	Range	Average	Standard deviation
Wind speed	WS	(m/s)	0–5.1	0.5	1.19
Cloudiness	CL	(%)	0–100	3.55	14.35
Visibility	Vis	(%)	10–100	97.59	12.60
Temperature	Temp	Celsius (°C)	2.8–30	18.41	4.37
Night cooling	NC	Celsius (°C)	−1.9–6.1	1.88	1.80
Pluviosity	PLU	(1—low; 2—moderate; 3—heavy)	0–1	0.02	0.13
Type of pluviosity	TPLU	(1—fog; 2–rain; 3—hail; 4—snow)	0–2	0.02	0.20
Humidity	Hum	(%)	0–100	66.15	16.94
Altitude	Alt	Metres (m)	357–1978	1078.28	429.07
Vegetation structure	VegStr	(1—herbaceous; 2—brushwood; 3—open forests 4—young forests; 5—mature dense forests)	1–5	3.32	1.56
Tree Layer (>2 m)	TreeL	(%)	0–95	41.50	33.65
Shrub Layer (0.3–2 m)	ShrubL	(%)	0–98	14.71	15.94
Herbaceous Layer (0–0.3 m)	HerbL	(%)	0–90	26.11	23.26
Vegetation cover	TotalVeg	(%)	30–98	78.18	20.35

*Note*: The climatic variables—wind speed, cloudiness, visibility, temperature and night cooling. The landscape variables, excluding the altitude, (vegetation structure, tree layer, shrub layer, herbaceous layer and vegetation cover) were measured within a 200 m radius around the sampling site. The classification of the vegetation structure was determined based on the average vegetation height as proposed by Martí and del Moral (2003): herbaceous (grasslands, prairies, swards, etc.), brushwood (with a great diversity of nonwoody medium shrubs), open forest, young forest (dense forest with tree layer height <12 m) and mature dense forest (tree layer >12 m).

Bat capture and handling followed all relevant guidelines and regulations and was approved by the Ethical committee at the ICNF (Instituto da Conservação da Natureza e das Florestas). This study was also carried out in compliance with the ARRIVE guidelines (https://arriveguidelines.org/).

### 2.4 Statistical analysis

#### 2.4.1 Environmental conditions shaping bat assemblages and bat species association with seasonal ecological assemblages

**FIGURE 3:**
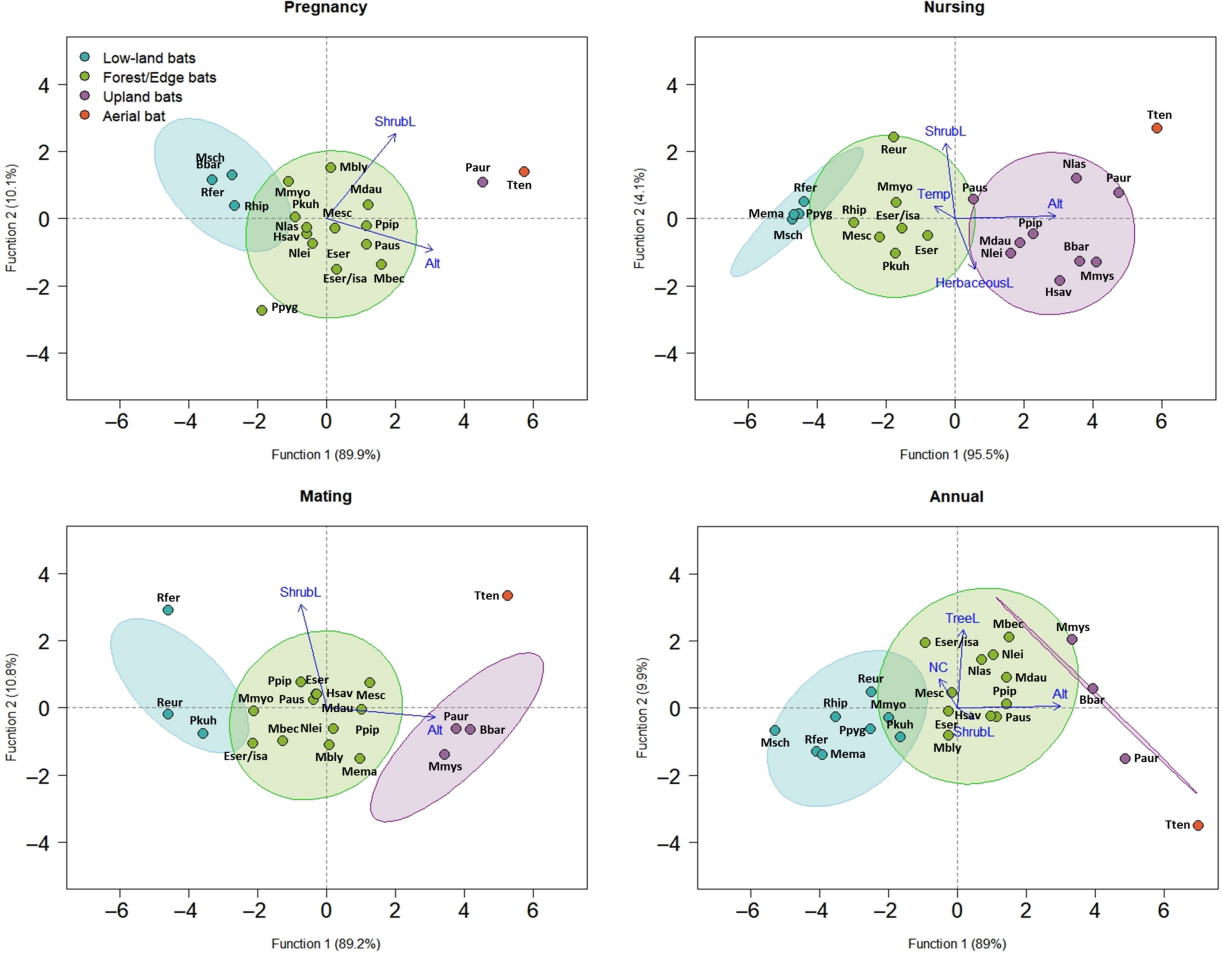
Territorial map with the position of each bat species and their ecological assemblages along the two significant discriminant functions during each season of the bats' active biological cycle, and on the entire (annual) cycle. Activity seasons were as follows: pregnancy (May/June), nursing (July/August) and mating (September/October). The direction of the relevant variables is indicated by the direction of the arrows and their relevance by their respective length (Alt, altitude; HerbaceousL, herbaceous layer; NC, night cooling; ShrubL, shrubs layer; Temp, temperature; TreeL, tree layer). See the meaning of species acronyms in Table 2.

Using a hierarchical cluster analysis with squared Euclidean distance as a dissimilarity measure, bat species were grouped into four different ecological assemblages based on their weighted preference for several biophysical variables over the year (Maroco, 2010). This analysis also allowed us to identify global characteristics and main drivers of each assemblage. The names proposed for each assemblage were related to their habitat preferences, namely mosaic bats, forest/edge bats, upland bats and aerial bats, see results section (Figure 3, Table 1; Appendix S1: Tables S1–S4, Figure S2–S5). Analyses were performed in the ‘Cluster’ R package. A dendrogram was calculated for the cluster analysis using the ‘factoextra‘ and ‘dendextend’ R packages. R‐squared metrics were used as retention criteria for the number of clusters (Maroco, 2010). The option with fewer clusters and a higher fraction of explained variance (closest to 80%) was refined with a nonhierarchical k‐Means test. An ANOVA was computed to identify which variables had higher importance for the retained clusters (Maroco, 2010). Another hierarchical cluster analysis was performed to group bat species into ecological assemblages for each season of their biological cycle, to identify the most relevant variables of each season with the weighted average of several biophysical variables for each season.

To identify which variables better discriminate bat ecological assemblages throughout the year extracted from the cluster analysis, a linear discriminant analysis (LDA) was computed with the method of Wilks' Λ, using the ‘MASS’ R package. The result of the discriminant analysis was visualized with the R packages ‘devtools ‘and ‘ggord’. Finally, we used the output of classification statistics to obtain the classification functions (Maroco, 2010).

#### 2.4.2 Bat species vulnerability

To assess species' vulnerability to changing environmental conditions, species marginality was computed for each environmental variable identified as significant by the annual discriminant analysis (Hirzel et al., 2002). This analysis provided a global overview of species vulnerability and its seasonality for the significant variables of each biological season. Species marginality was graphically correlated with the weighted average of the same variables (Appendix S1: Table S22). To calculate the weighted average, the number of individuals in each sampling was multiplied by the value of the concerned variable and summed all of these products for each species. Then, the sum of all of these products was divided by the number of individuals of the species. Climate change was considered to affect each variable in a specific direction, which allowed us to infer the main direction of the threat, that is, which side of the niche space is more likely to disappear in the future. For example, we assumed that areas with a higher level of forest cover face a higher risk of being lost than areas of low forest cover mainly due to wildfires, droughts, forest encroachment and pests' outbreaks (Frick et al., 2020; Gonçalves & Sousa, 2017). Similarly, bat species strongly associated with high‐altitude conditions were considered particularly vulnerable as high‐mountain habitats, and climatic conditions tend to disappear faster as warmer climatic envelopes shift to higher altitudes due to climate change (Engler et al., 2011). On the contrary, species associated with high percentage of shrub cover could be less threatened because the number of wildfires has increased thus promoting an increase in the area of shrubs (Mirts et al., 2022). Wildfire episodes are likely to increase according to predicted climate change (Goss et al., 2020).

Finally, our assessment of the species' vulnerability to environmental changes was compared with the national conservation status of each bat species (Appendix S1: Table S21).

## 3 RESULTS

### 3.1 Environmental conditions shaping bat assemblages

We captured 1035 bats belonging to 23 species (Table 2, Appendix S1: Table S22). We carried out 37 sampling visits during pregnancy season with an average of 6.97 ± 8.86 bat captures (Mean ± SD), 51 samplings during nursing and 39 during mating season with an average of 10.29 ± 13.66 and 6.46 ± 8.82 of bats captured (Mean ± SD), respectively. The discriminant analysis extracted two discriminant functions for both pregnancy and mating seasons, retaining both seasons' altitude and shrub layer as statistically significant variables (Figure 3, Table 1, Appendix S1: Tables S10–S13, S18–S21). Function 1 was mainly defined by altitude, explaining 89.9% and 89.2% of the variability between groups, in pregnancy and mating seasons, respectively. Regarding the nursing season, the discriminant analysis extracted three discriminant functions, with temperature, altitude, shrub layer and herbaceous layer as statistically significant variables (Figure 3 Table 1, Appendix S1: Tables S14–S17). Function 1 was essentially defined by altitude and temperature, explaining 95.5% of the variability between groups. The annual discriminant analysis extracted three discriminant functions, retaining altitude, tree layer, shrub layer and night cooling as statistically significant variables for groups' discrimination (Figure 3, Appendix S1: Tables S1, S6–S9). Function 1 was essentially defined by altitude, explaining 89.0% of the variability between groups.

**TABLE 2:** Species sampled on each biological season and their ecological assemblage previously identified on hierarchical cluster analysis and discriminate analysis. The empty cells mean the species was not captured in this season.

Species	Acronym	Pregnancy		Nursing		Mating	
		Assemblage	Abundance	Assemblage	Abundance	Assemblage	Abundance
*Myotis mystacinus*	Mmys			Upland	5	Upland	2
*Myotis bechsteinii*	Mbec	Forest/Edge	2			Forest/Edge	1
*Eptesicus serotinus/isabellinus*	Eser/isa	Forest/Edge	2	Forest/Edge	5	Forest/Edge	2
*Nyctalus lasiopterus*	Nlas	Forest/Edge	3	Upland	1		
*Nyctalus leisleri*	Nlei	Forest/Edge	14	Upland	39	Forest/Edge	31
*Barbastella barbastellus*	Bbar	Mosaic	3	Upland	22	Upland	29
*Myotis daubentonii*	Mdau	Forest/Edge	13	Upland	34	Forest/Edge	15
*Rhinolophus euryale*	Reur			Forest/Edge	9	Mosaic	6
*Eptesicus serotinus*	Eser	Forest/Edge	9	Forest/Edge	24	Forest/Edge	8
*Pipistrellus kuhlii*	Pkuh	Forest/Edge	3	Forest/Edge	8	Mosaic	1
*Myotis blythii*	Mbly	Forest/Edge	2			Forest/Edge	1
*Pipistrellus pipistrellus*	Ppip	Forest/Edge	29	Upland	77	Forest/Edge	42
*Plecotus austriacus*	Paus	Forest/Edge	33	Upland	55	Forest/Edge	12
*Myotis escalerai*	Mesc	Forest/Edge	50	Forest Edge	80	Forest/Edge	27
*Hypsugo savii*	Hsav	Forest/Edge	14	Upland	45	Forest/Edge	21
*Pipistrellus pygmaeus*	Ppyg	Forest/Edge	1	Mosaic	1		
*Myotis myotis*	Mmyo	Forest/Edge	7	Forest/Edge	13	Forest/Edge	4
*Rhinolophus hipposideros*	Rhip	Mosaic	6	Forest/Edge	7		
*Miniopterus schreibersii*	Msch	Mosaic	2	Mosaic	4		
*Tadarida teniotis*	Tten	Aerial	4	Aerial	7	Aerial	5
*Plecotus auritus*	Paur	Upland	47	Upland	55	Upland	29
*Rhinolophus ferrumequinum*	Rfer	Mosaic	14	Mosaic	22	Mosaic	14
*Myotis emarginatus*	Mema			Mosaic	12	Forest/Edge	2

The number of assemblages remained consistent throughout the year (Appendix S1: Figures S1–S5). The characteristics that gave name to bat assemblages were as follows: upland and forest/edge bats were more related to areas at high altitude with high percentage of tree layer but in opposite weights for each group. The mosaic bats were more related to areas at low altitude with low night cooling, some forest coverage and medium‐high percentage of shrub cover. Aerial bats were very related to areas at high altitude with high percentage of shrubs and low percentage of tree cover (Table S7).

Regarding the annual analyses, eight species were grouped into an ecological assemblage denominated as mosaic bats. The second group was named aerial bats and was constituted by only one species. The third group clustered together the three upland bats' species. Finally, the fourth group included the forest/edge bats being constituted by 11 species (Figure S1). However, the species composition of each assemblage changed throughout the seasons, revealing different levels of species' ecological plasticity. During pregnancy, the mosaic bats were constituted by four species, the aerial bats and upland bats by one, and the forest/edge bats by 14. In the nursing season, the mosaic bats had four species, the aerial bats one species, upland bats nine species and forest/edges bats had seven species. For the mating season, the mosaic bats had three species, upland bats had three species, the aerial bats had one species, and forest/edges bats had 12 species (Figure 3 and Table 2).

The territorial map (Figure 3; designation of the discriminant analysis plot between the two main explanatory functions) shows the position of each bat species and respective ecological assemblage as well as the scores of the two main discriminant functions by season and annually. The results of the classification statistics showed that all bat species were classified correctly (100% of classification in all discriminant analyses; Appendix S1: Tables S7, S11, S15, S19).

### 3.2 Bat species association with seasonal ecological assemblages

Bats exhibited different ecological preferences along their biological cycle, resulting in different bat assemblages throughout the year (Figure 3). During pregnancy, the number of bat species associated with the forest/edge assemblage (14 species) and mosaic bats (four species) increased while upland (one species) decreased compared with mating season (Table 2 and Appendix S1: Table S10). During nursing, the highest number of bat species grouped at the upland bats' assemblage (9 species) (Table 2 and Appendix S1: Table S14) while during mating bat preferences changed again towards the forest/edge assemblage (12 species; Table 2 and Appendix S1: Table S18). The aerial bat assemblage remained constant in terms of species richness and composition throughout the year mainly because is represented by only one species (*Tadarida teniotis*).

Other bat species were also exclusively associated with a single ecological assemblage, namely *R. ferrumequinum* and *M. schreibersii* (mosaic bats), *M. myotis*, *M. blythii*, *M. escalerai*, *M. bechsteinii*, *E. serotinus*, *E. serotinus*/*isabellinus* (forest/edge bats), and *P. auritus* (upland bats). The other species had dynamic biophysical preferences, occurring across different assemblages throughout the year (Table 2). However, no species integrated more than two different bat assemblages (Table 2).

### 3.3 Bat species vulnerability

**FIGURE 4:**
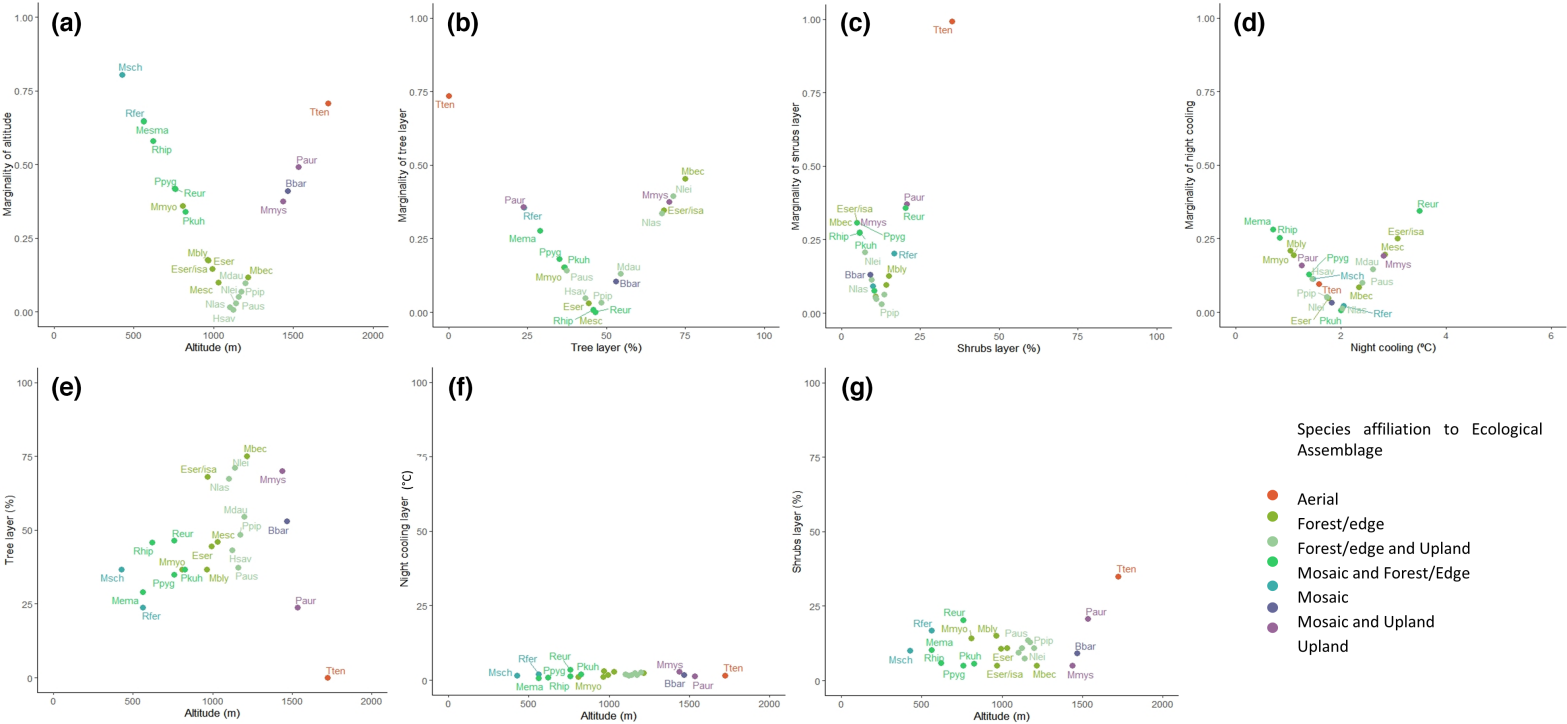
Marginality of bats' niche usage weighted by their environmental preferences as assessed on the annual discriminant analysis: (a) Marginality regarding altitude, (b) marginality regarding tree cover, (c) marginality regarding shrub cover and (d) marginality regarding night cooling. (e) Relationship between bat species and their assemblages and the weighted average of tree cover and altitude variables, (f) relationship between bat species and their assemblages and the weighted average of night cooling and altitude variables and (g) relationship between bat species and their assemblages and the weighted average of shrub cover and altitude variables. The colours represent the species' affiliation with the ecological assemblages throughout the year. The intermediate colours between assemblages mean the occurrence of the species in different assemblages. Please see each species affiliation in each season and the meaning of species acronyms in Table 2.

Taking into account species marginality and the respective weighted average for each variable, we considered that the most vulnerable species were associated with habitats at high altitude with a high proportion of tree layer cover, an intense night cooling and low proportion of shrubs (Figure 4; Appendix S1: Table S5). Regarding altitude and tree layer, the most vulnerable bat species were *M. mystacinus*, from the upland bats' assemblage, and *M. bechsteinii*, from the forest/edge bats' assemblage (Figure 4, Table 2). Regarding the shrubs layer and altitude, *M. mystacinus* and *M. bechsteinii* were the most vulnerable species associated for both of their assemblages (upland and forest/edge, respectively) (Figure 4). However, between night cooling and altitude, species just expressed a relevant variation across the altitudinal axe (Figure 4).

*R. euryale* was strongly associated with night cooling, thus being most vulnerable to warmer nights, while *M. bechsteinii, P. pygmaeus, M. mystacinus and E. serotinus/isabellinus* were the most associated with reduced shrub cover (Figure 4).

The effect of each variable on bat ecological preferences varied considerably throughout the year, which might reflect their vulnerability to future biophysical changes. Species marginality which in turn assesses the vulnerability varied along the bat's biological cycle. During pregnancy, *P. auritus* and *T. teniotis* were the most vulnerable species to the loss of high‐altitude habitats, while *P. pygmaeus* was particularly vulnerable due to its association sites with reduced shrub cover (Figure 4). During nursing, *P. auritus*, *T. teniotis*, *M. mystacinus* and *B. barbastellus* were the most vulnerable species due to their association with high‐altitude habitats, while *P. kuhlii* and *R. hipposideros* were more associated with areas of low shrub cover. The *H. savii* had a higher association with high herbaceous cover (Appendix S1: Figure S7). During mating, *P. auritus*, *T. teniotis* and *B. barbastellus* were the most vulnerable species due to association with high‐altitude habitats, while *M. emarginatus* and *E. serotinus/isabellinus* were more related to habitats with a low shrub cover (Appendix S1: Figure S8).

## 4 DISCUSSION

We show that bat community composition is highly conditioned by the altitudinal gradient and to a lower degree by shrub and tree cover. According to their biophysical characteristics, we identified four main species assemblages': aerial bats, forest/edge bats, mosaic bats and upland bats. These four assemblages were statistically consistent across the year, although several species change between ecological assemblages throughout the year. These dynamics in the composition of bat assemblages seem to allow species to coexist by taking advantage of the temporal variations in available resources. The complementarity between annual and seasonal analysis allowed us to identify the main assemblages and their global characteristics with annual analysis and their composition changes across the seasons with seasonal analysis. According to the species marginality in relation to the available biophysical environment and the direction of current environmental threats, upland bats stand out as the most vulnerable assemblage to foreseeable environmental changes, especially *M. mystacinus*. Bat assemblages associated with high‐altitude conditions, mature forests (as shown by the annual analyses) and a reduced level of shrub cover were also associated with greater vulnerability. However, species vulnerability is also related to their specialization to biophysical conditions (Piksa, 2008), since species that are restricted to a single ecological assemblage (i.e. low environmental plasticity) are likely to be more vulnerable to environmental change.

### 4.1 Environmental conditions shaping bat assemblages

It is well known that seasonal changes can shape bat community structure (Stevens, 2013). However, our results showed that altitude was the strongest driver of bat community composition, followed by shrub and tree cover. The effect of altitude is probably associated with climatic conditions, although our measured weather variables were not significant (temperature, humidity, wind speed, pluviosity, type of pluviosity and cloudiness). This is probably because bats took advantage of the environmental resources in locations and periods in which the weather conditions were more favourable for bat activity. So, several of the mentioned weather variables remained with low variation throughout the seasons such as the near absence of pluviosity. Therefore, no significant differences between species allow them to be discriminated by these variables. Consequently, species that are known to be associated with colder conditions, like *B. barbastellus*, *P. auritus* and *M. mystacinus*, were associated with higher altitudes (Rebelo et al., 2010; Widerin & Reiter, 2017). The altitudinal gradient was the best predictor of bat assemblage in all seasons of our models, likely because it is also associated with a great diversity of habitats created by the existing climate gradient (Jansen & Correia, 2002). The shrub cover showed a relevant influence on bats in all seasons, although less relevant than that of altitude. Additionally, other biophysical characteristics like temperature and herbaceous cover also seem to have influenced bat community composition during the nursing season. A likely consequence of the particular demands of this biological season.

Throughout the year, aerial bats use crevices as roosts and forage at exceptionally high altitudes (approximately 1600 m above ground level; O'Mara et al., 2021); thus, there are likely more natural roosts in areas with cliffs at higher altitudes and with high shrub cover and very low tree cover (Marques et al., 2004). The species composition of the upland bats varied between seasons, probably taking advantage of a higher abundance of invertebrates in high‐altitude habitats during the nursing season in response to higher temperatures (Lara‐Romero et al., 2019; Mata et al., 2016). Forest/edge bats were found in habitats at medium altitudes and medium‐high tree and shrub cover. These conditions seem particularly important during the pregnancy and mating seasons when milder climates are present in forest habitats (roosts, prey, etc.). During mating, some species may select habitats at higher altitudes where they may find higher prey availability (Beilke et al., 2021; Parsons & Jones, 2003; Russ & Montgomery, 2002). The preference shown by mosaic bats for lower habitats with medium‐high shrub cover and medium‐low tree cover during pregnancy and nursing seems less evident during the mating season. During the mating season, species such as *M. emarginatus* seem to be able to explore habitats at higher altitudes.

### 4.2 Bat species association with seasonal ecological assemblages

Our results demonstrate that some species remain in the same ecological assemblage throughout the year, perhaps due to permanent species‐specific environmental requirements such as proximity to roosts (e.g. caves for *R. ferrumequinum* and *M. schreibersii*) or specific habitats (mature forests for *M. mystacinus* and *M. bechsteinii*; Dietz et al., 2009; Piksa et al., 2011). In contrast, some species track variable resources (e.g. prey availability), thus integrating different assemblages throughout the year as suggested by Lara‐Romero et al. (2019). This seems to be the case of species like *M. daubentonii*, *P. pipistrellus*, *N. leisleri* and *N. lasiopterus* (Dietz et al., 2009; Popa‐Lisseanu et al., 2009).

During pregnancy, mid‐altitude habitats were used by a great number of species. Previous studies found that milder conditions and less human disturbance reduce energy losses during a demanding season for females (Lintott et al., 2014). During nursing, the movement of several species to the high altitudes associated with upland bats assemblage shows the occurrence of favourable conditions and resources during the summer at higher altitudes (e.g. prey availability) that may sustain parental care (Adams & Hayes, 2008; Womack et al., 2013). Additionally, mid‐ and high‐altitude habitats were also selected during mating and pre‐hibernation activities (McGuire et al., 2013).

Our results suggest that sorting species into groups based on one/few static variables, for example, echolocation (Neuweiler, 1989), diet or yearly habitat use (Estrada‐Villegas et al., 2012; Kalko & Handley, 2001; Oelbaum et al., 2019), may overlook important aspects of the ecological dynamics of bats. We therefore highlight that ecological assemblages may also present temporal dynamics that have significant influence when studying community functioning and bat vulnerability to climate change stressors.

### 4.3 Bat species vulnerability

Our study suggests that upland and forest/edge bats are particularly vulnerable to ongoing environmental changes due to their strong association with higher altitudes, and secondarily to mountain shrubs, and forest, as shown by the annual analyses. These habitats are highly threatened by anthropogenic impacts such as severe wildfires, droughts, and/or long‐term climate change (Ancillotto et al., 2021; Blakey et al., 2019; Bravo et al., 2008; Schmeller et al., 2022). On the contrary, it was visible some association with shrubs by forest/edge and upland bats (the last one with lesser importance), in part, related to specific endemics shrubs habitats occurring at the highest altitudes but also due to habitat change as a result of climate change. Climate change indirectly affects vegetation due to the increase in frequency and intensity of forest wildfires and droughts, which together with deforestation, and agricultural abandonment at lower altitudes, leads to accelerated habitat loss (Jones et al., 2009). As a consequence, the substitution of mature forests by extensive areas of early successional shrublands is increasing (Mirts et al., 2022). The association of several species to shrubs may also demonstrate that some of them have some level of capacity to adapt to habitat changes. On the contrary, some species seem to be quite species‐specific and may not adapt so well to habitat changes, especially for those bat species that are strongly associated with forest habitats, avoiding areas with a high percentage of shrub cover. Furthermore, high‐mountain habitats, apart from being restricted to a limited geographic area, are currently under great pressure from climate change due to the migration of low‐mountain habitats and species towards mountain tops (Bravo et al., 2008; Kohler & Maselli, 2009).

Our study identified the upland bat *M. mystacinus* as the most vulnerable species in the region due to its permanent association with high‐mountain forested habitats. This species has already been acknowledged as one of the European species most threatened by climate change (McGowan et al., 2021; Rebelo et al., 2010). Moreover, *M. bechsteinii* (forest/edge bats) is known to be a sedentary species (Napal et al., 2013) and very restricted to the mid‐altitude forest, making it the second most vulnerable bat species in the region.

Importantly, we found that the high level of vulnerability of these species is not fully reflected on their national conservation status. Indeed, *M. mystacinus, M. blythii, P. austriacus and M. escalerai* are the only species in our study with a threat status (Portuguese conservation status: Vulnerable, Critically Endangered, Near Threatened and Vulnerable, respectively), while all other species are classified as Data Deficient or Least Concern in the Portuguese mammals' red book (Mathias et al., 2023). While this discrepancy is expected and understandable, given the well‐defined criteria for determining species threat status (e.g. population decline and distribution contractions), it also reveals that obtaining such data may not provide timely responses for effective conservation actions (Hannah, 2012; Rinnan & Lawler, 2019), especially for regional assessments. In that respect, our approach be a useful complementary tool to anticipate specific conservation threats. This is particularly relevant as the changes in these populations' size and distribution might be too quick for being perceived in national threat status valuations before being too late.

## 5 LIMITATIONS

Our analysis did not cover the entire bat life cycle, as we have not sampled during the hibernation period. Instead, we sampled during bat active periods that are strongly associated with survival and reproduction (Sherwin et al., 2013).

The biophysical conditions included in this study are unlikely to represent all relevant drivers of bat niche differentiation (Kooyers et al., 2017), which include other factors, such as prey availability (Chowdhury et al., 2020; Rydell et al., 2010; Wray et al., 2021). Bats' behaviour, for example during swarming or migration, can also present other drivers of community dynamics (Caprio et al., 2020; Piksa, 2008; Piksa et al., 2011). Moreover, little is known about the drivers of altitudinal bat migrations, for instance, if species perform regional or long‐distance movements (McGuire et al., 2013). Although our data suggest that there is an indication of seasonal altitudinal movements in several species, more studies are needed to clarify this subject (e.g. by employing a biologging approach).

The shrub layer was relatively important throughout the seasons (~10%). However, this variable needs more study to understand its importance for bat species, in particular on bats' relationship to diversity of shrubs habitats. We believe that the high diversity of shrub habitats in the study area (Jansen & Correia, 2002) could be, in part, the justification for the bat preferences because many of these habitats host a relevant diversity of insects (prey availability) (Jansen & Correia, 2002). We emphasize the importance of making detailed characterization of habitats along the different successional stages.

The use of nonsystematic samplings (25.4%) in the methodological approach had some implications, particularly in bat species' seasonal preferences that did not cover completely all of the habitats' seasonality. Yet, in our situation, a sampling effort design in accordance with the proportion of each habitat would have likely overlooked underrepresented microhabitats such as ponds, streams, and small forest patches that are acknowledged to be relevant for bat diversity, especially for rarer species. In addition, using mist nets to sample bats has some known biases, for example, some species are very difficult to capture because they fly very high or because they can detect and avoid the nets (MacSwiney et al., 2008; O'Mara et al., 2021). Yet, the great number of species captured for this study (23 out of the 27 bat species given for Portugal) shows that these limitations are likely to have a low impact on our results.

Finally, the *E. serotinus/isabellinus* species complex stands out from our results by its high level of vulnerability. The fact that the study area coincides with the contact zone of these cryptic species, and their high probability of hybridization (Centeno‐Cuadros et al., 2019) renders the interpretation of these results particularly difficult for this species complex.

## 6 CONCLUSION

Here, we show that the gradient associated with altitude is the main driver of bat community composition with important differences between seasons. The shrub and tree cover showed a minor but also significant contribution. While some bat species are restricted to a single ecological assemblage, others have shown greater plasticity throughout the year, taking advantage of temporal variations of resources. This has direct implications to species vulnerability to environmental changes where species associated solely with high‐mountain habitats and forests may be under greater pressure.

## AUTHOR CONTRIBUTIONS

**Helena Raposeira:** Conceptualization (equal); data curation (equal); formal analysis (lead); funding acquisition (equal); investigation (lead); methodology (lead); resources (lead); software (lead); supervision (equal); validation (equal); visualization (lead); writing – original draft (lead); writing – review and editing (lead). **Pedro Horta:** Conceptualization (equal); data curation (equal); methodology (equal); resources (equal); validation (equal); writing – original draft (supporting); writing – review and editing (supporting). **Ruben Heleno:** Conceptualization (equal); methodology (equal); supervision (equal); validation (equal); writing – original draft (supporting); writing – review and editing (supporting). **Hugo Rebelo:** Conceptualization (equal); funding acquisition (equal); methodology (equal); resources (equal); supervision (equal); validation (equal); writing – original draft (supporting); writing – review and editing (supporting).

## FUNDING INFORMATION

H. Rap. was funded by Portuguese Foundation for Science and Technology (FCT) with a Ph.D. grant (SFRH/BD/124367/2016) and by Bat Conservation International with a Student Scholarship (grant number SS1805). P. Hor. was funded by FCT with a PhD grant (SFRH/BD/117979/2016). H. Reb. and R.H. were also funded by FCT through grants DL57/2016/EEC2018/07 and UID/BIA/04004/2020, respectively. This research was supported under the scope of the FCT project PTDC/BIA‐ECO/31731/2017.

## CONFLICT OF INTEREST STATEMENT

All authors declare no competing interests.

## Supporting information


Appendix S1

Data S1

## Data Availability

The authors compromise to publish the dataset analysed in this manuscript in a publicly accessible repository after its acceptance.
