# Supplementary material for: Changing with the times: Seasonal environmental gradients unveil dynamic bat assemblages and vulnerability
Source: Ecol Evol. 2023 Jul 17;13(7):e10246. doi: 10.1002/ece3.10246 (PMC10352094; doi:10.1002/ece3.10246)
Supplement: Supplementary file 1 — Appendix S1 [file ECE3-13-e10246-s001.docx]

**Supplementary Information for**

Changing with the times: Seasonal environmental gradients unveil dynamic bat assemblages and vulnerability

Helena Raposeira*, Pedro Horta, Ruben Heleno, Hugo Rebelo

*Corresponding author: helena.raposeira@cibio.up.pt

**This PDF file includes:**

Supplementary text: Materials and Methods

Supplementary text: Results

Figs. S1 to S10

Tables S1 to S22

**Supplementary text: Materials and Methods**

In the discriminant analyses, the assumptions of normality and homogeneity of variance-covariance matrices of each group were tested with Kolmogorov-Smirnov test and with the Box M test, respectively. In the annual analysis, CL, Vis, Temp, Hum, PLU, TPLU, HerbaceousL, VegStr and TotalVeg (Table S1) were eliminated due to the lack of normality. According to the Box M test, the assumption of homogeneity of variance-covariance matrices was not valid (*M* = 30.700, *F* (10; 1065.418) = 2,231, *p* = 0.014). However, the discriminant analysis is robust to assumption violations, as long as the dimension of the minor group will be superior to the number of variables in the study and on which the group averages will not be proportional to your variances (Maroco, 2010). The variable WS was also excluded from the analysis because its effect was not significative and NC was maintained because had marginally significant (p<0.1). According to seasonal analysis, in the pregnancy season CL, WS, Vis, PLU, TPLU, HerbaceousL and TotalVeg were eliminated from the analysis because they didn’t comply with the assumption of normality. The Box M test, the assumption of homogeneity of variance-covariance matrices was valid (*M* = 7.738, *F* (3; 405.363) = 1.912; *p* = 0.127). The variables Temp, Hum, TreeL, NC and VegStr were also excluded from the analysis because they were not significant (Table S1). In the nursing season, CL, PLU, TPLU, Vis and VegStr were removed from the analysis due to the lack of normality. According to the Box M test, the assumption of homogeneity of variance-covariance matrices was valid (*M* = 19.404, *F* (10; 786.795) = 1.303; *p* = 0.224). The variables WS, Hum, TreeL, TotalVeg and NC were also excluded from the analysis because they were not significant (Table S1). With respect to the mating season, WS, CL, PLU, TPLU, Vis, VegStr and TotalVeg were removed from the analysis due to the lack of normality. Regarding to the Box M test, the assumption of homogeneity of variance-covariance matrices was valid (*M* = 12.521, *F* (6; 256.907) = 1.266; *p* = 0.273). Finally, Temp, Hum, TreeL, HerbaceousL and NC were also excluded from the analysis because they were not significant (Table S1).

**Supplementary text: Results**

*Environmental conditions shaping bat assemblages*

In the discriminant analysis, the functions 1 significantly discriminate the four groups during pregnancy (Λ=0.09; X_2(6)_ =38.15; p<0.001) and mating (Λ=0.05; X_2(6)_ =44.24 p<0.001). The second function are mainly defined by the presence of a shrub layer and also significantly discriminates the four groups during the pregnancy (Λ=0.611; X_2(2)_ =7.87; p<0.05), and mating seasons (Λ=0.50; X_2(2)_ =10.56; p<0.05).

Regarding the nursing season, the function 1 significantly discriminates four groups (Λ=0.05; X_2(12)_ =49.25; p<0.001). The second (Λ=0.62; X_2(6)_ =7.6; p=0.268) and third (Λ=0.96; X_2(2)_ =0.70; p=0.705) retained functions are mainly defined by the shrub and herbaceous layers. However, these functions do not significantly discriminate the four bat assemblages.

In the annual discriminant analysis, the function 1 significantly discriminates the four bat assemblages (Λ=0.04; X_2(12)_ =57.76; p<0.001). The second (Λ=0.43; X_2(6)_ =15.19; p<0.05) and third (Λ=0.89; X_2(2)_ =2.08; p=0.35) retained functions were defined by tree layer, shrub layer and night cooling. However, the second function significantly discriminates the four assemblages, unlike the third function.

*Bat species association to seasonal ecological assemblages*

*Cluster analysis*


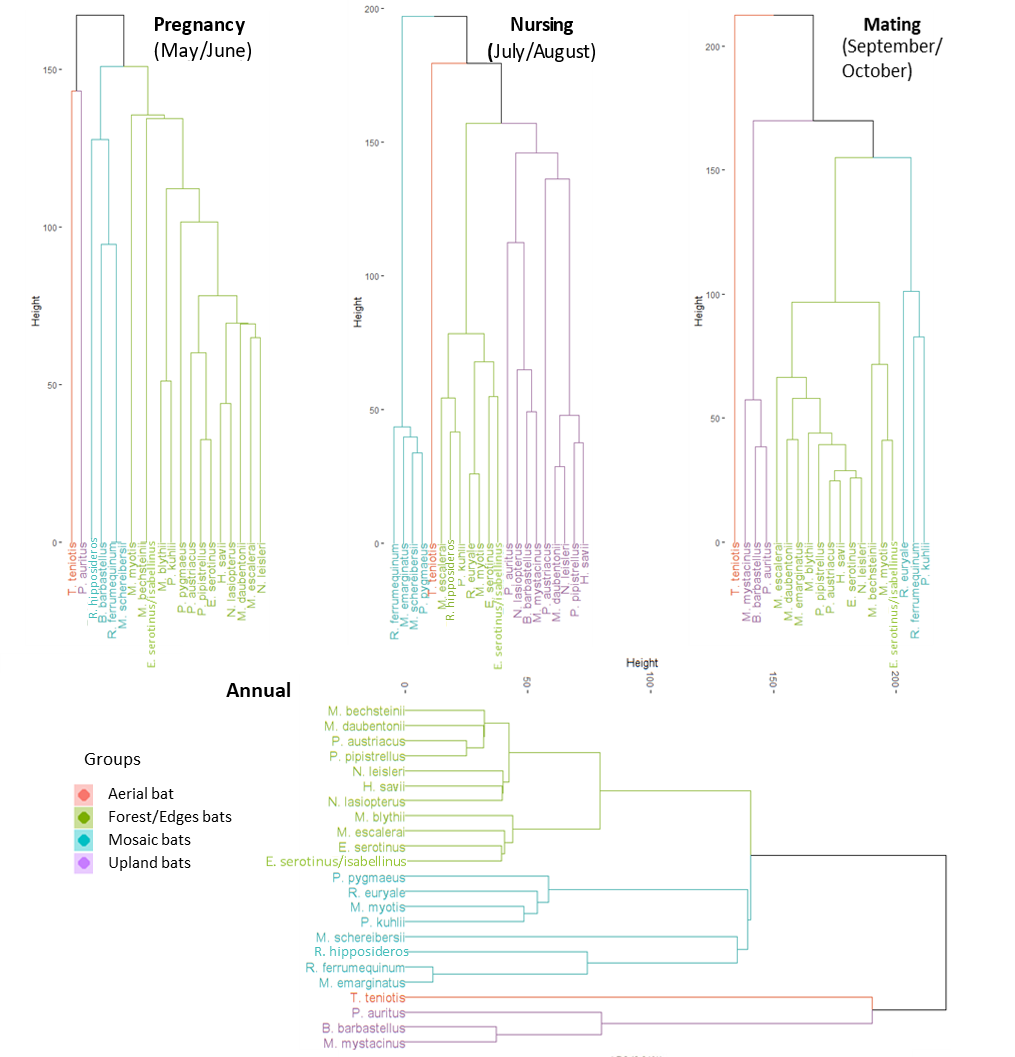


Fig. S1 - Hierarchical cluster dendrograms, using single linkage and squared Euclidean distance, represent bat assemblages according to their biophysical preferences during an annual cycle and in each biological season (May/June – pregnancy; July/August – nursing; September/October – mating).

*Annual*

Table S1 – Bat classification, of annual analysis, by K-mean methods with K=4 and F statistics to each dimension. Biophysical variables are available in table 1. Significant values are shown in bold.

| ANOVA | | | | | | |
| --- | --- | --- | --- | --- | --- | --- |
|  | **Cluster** | | **Error** | | **F** | **Sig.** |
|  | **Mean Square** | **df** | **Mean Square** | **Df** |  |  |
| WS | 0.084 | 3 | 0.223 | 19 | 0.375 | 0.772 |
| CL | 0.565 | 3 | 0.512 | 19 | 1.104 | 0.372 |
| Vis | 6.177 | 3 | 5.907 | 19 | 1.046 | 0.395 |
| Temp | 7.162 | 3 | 3.223 | 19 | 2.222 | 0.119 |
| PLU | 0.000 | 3 | 0.000 | 19 | 1.008 | 0.411 |
| TPLU | 0.000 | 3 | 0.000 | 19 | 1.008 | 0.411 |
| Hum | 142.157 | 3 | 60.874 | 19 | 2.335 | 0.106 |
| **NC** | 1.433 | 3 | 0.563 | 19 | 2.547 | **0.086** |
| **Alt** | 730714.398 | 3 | 12421.943 | 19 | 58.824 | **<0.001** |
| **VegStr** | 1.453 | 3 | 0.461 | 19 | 3.151 | **0.049** |
| **TreeL** | 1181.761 | 3 | 184.062 | 19 | 6.420 | **0.003** |
| **ShrubL** | 190.699 | 3 | 24.291 | 19 | 7.851 | **0.001** |
| HerbaceousL | 91.995 | 3 | 185.063 | 19 | 0.497 | 0.689 |
| **TotalVeg** | 810.418 | 3 | 189.669 | 19 | 4.273 | **0.018** |
| The F tests should be used only for descriptive purposes because the clusters have been chosen to maximize the differences among cases in different clusters. The observed significance levels are not corrected and thus cannot be interpreted as tests of the hypothesis that the cluster means are equal. | | | | | | |


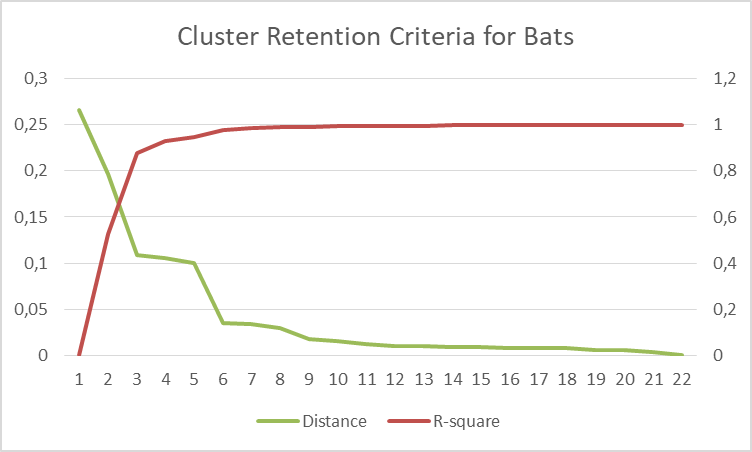


Fig. S2 – Intersection of distance between standardized bat clusters (between 0 and 1) and R- squared of each cluster on the annual analysis.

*Pregnancy season*

Table S2 – Bats classification, of pregnancy season analysis, by K-mean methods with K=4 and F statistics to each dimension. Biophysical variables are available in table 1. Significant values are shown in bold.

| ANOVA | | | | | | |
| --- | --- | --- | --- | --- | --- | --- |
|  | **Cluster** | | **Error** | | **F** | **Sig.** |
|  | **Mean Square** | **df** | **Mean Square** | **Df** |  |  |
| WS | 0.578 | 3 | .692 | 16 | 0.836 | 0.494 |
| CL | 2.978 | 3 | .996 | 16 | 2.990 | 0.062 |
| Vis | 3.356 | 3 | 5.903 | 16 | 0.569 | 0.644 |
| Temp | 1.433 | 3 | 5.873 | 16 | 0.244 | 0.864 |
| PLU | 0.001 | 3 | .001 | 16 | 2.358 | 0.110 |
| TPLU | 0.005 | 3 | .002 | 16 | 2.281 | 0.118 |
| Hum | 182.880 | 3 | 223.361 | 16 | 0.819 | 0.502 |
| **Alt** | 809195.557 | 3 | 12230.963 | 16 | 66.160 | **<0.001** |
| VegStr | 1.817 | 3 | 1.268 | 16 | 1.433 | 0.270 |
| TreeL | 1454.943 | 3 | 516.744 | 16 | 2.816 | 0.072 |
| **ShrubL** | 339.241 | 3 | 21.557 | 16 | 15.737 | **<0.001** |
| HerbaceousL | 412.516 | 3 | 477.962 | 16 | 0.863 | 0.480 |
| **TotalVeg** | 1526.666 | 3 | 185.584 | 16 | 8.226 | **0.002** |
| **NC** | 4.702 | 3 | 1.209 | 16 | 3.888 | **0.029** |
| The F tests should be used only for descriptive purposes because the clusters have been chosen to maximize the differences among cases in different clusters. The observed significance levels are not corrected for this and thus cannot be interpreted as tests of the hypothesis that the cluster means are equal. | | | | | | |


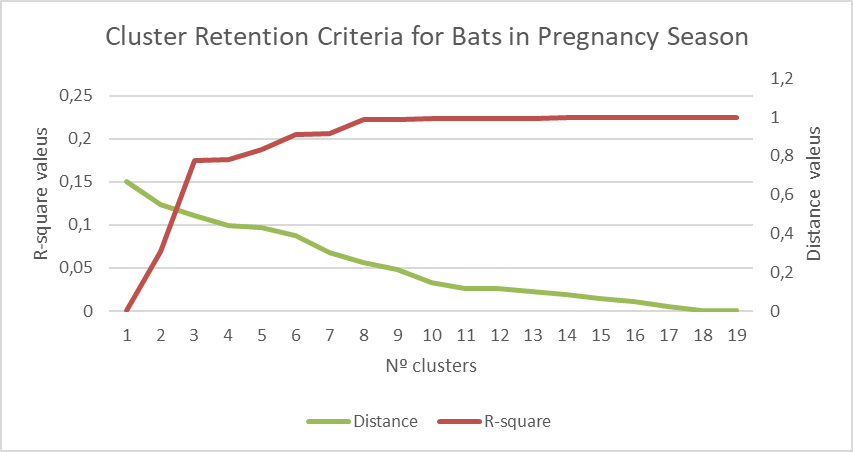


Fig. S3 – Intersection of distance between bat clusters relativized (between 0 and 1) and R- squared of each cluster, of pregnancy analysis.

*Nursing season*

Table S3 – Bats classification, of nursing season analysis, by K-mean methods with K=4 and F statistics to each dimension. Biophysical variables are available in table 1. Significant values are shown in bold.

| ANOVA | | | | | | |
| --- | --- | --- | --- | --- | --- | --- |
|  | **Cluster** | | **Error** | | **F** | **Sig.** |
|  | **Mean Square** | **df** | **Mean Square** | **Df** |  |  |
| WS | 0.545 | 3 | 0.266 | 17 | 2.046 | 0.146 |
| CL | 0.071 | 3 | 0.093 | 17 | 0.758 | 0.533 |
| Vis | 5.913 | 3 | 6.459 | 17 | 0.915 | 0.454 |
| **Temp** | 11.142 | 3 | 2.798 | 17 | 3.983 | **0.026** |
| **Hum** | 186.433 | 3 | 40.188 | 17 | 4.639 | **0.015** |
| **Alt** | 963173.188 | 3 | 11906.791 | 17 | 80.893 | **<0.001** |
| VegStr | 1.663 | 3 | 0.645 | 17 | 2.579 | 0.088 |
| TreeL | 1104.980 | 3 | 558.188 | 17 | 1.980 | 0.155 |
| **ShrubL** | 210.072 | 3 | 47.825 | 17 | 4.393 | **0.018** |
| **HerbaceousL** | 162.809 | 3 | 59.506 | 17 | 2.736 | **0.076** |
| TotalVeg | 727.441 | 3 | 308.517 | 17 | 2.358 | 0.108 |
| NC | 3.290 | 3 | 1.813 | 17 | 1.814 | 0.183 |
| The F tests should be used only for descriptive purposes because the clusters have been chosen to maximize the differences among cases in different clusters. The observed significance levels are not corrected for this and thus cannot be interpreted as tests of the hypothesis that the cluster means are equal. | | | | | | |


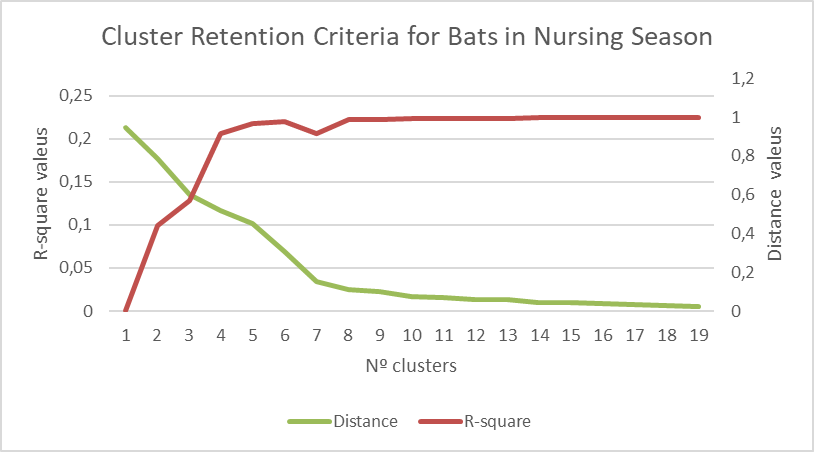


Fig. S4 – Intersection of distance between bat clusters relativized (between 0 and 1) and R- squared of each cluster, of nursing analysis.

*Mating season*

Table S4 – Bats classification, of mating season analysis, by K-mean methods with K=4 and F statistics to each dimension. Biophysical variables are available in table 1. Significant values are shown in bold.

| ANOVA | | | | | | |
| --- | --- | --- | --- | --- | --- | --- |
|  | **Cluster** | | **Error** | | **F** | **Sig.** |
|  | **Mean Square** | **df** | **Mean Square** | **Df** |  |  |
| WS | 0.386 | 3 | 0.292 | 15 | 1.324 | 0.304 |
| CL | 0.644 | 3 | 0.925 | 15 | 0.696 | 0.569 |
| Vis | 19.383 | 3 | 73.656 | 15 | 0.263 | 0.851 |
| Temp | 6.225 | 3 | 3.352 | 15 | 1.857 | 0.180 |
| Hum | 181.274 | 3 | 105.024 | 15 | 1.726 | 0.205 |
| **Alt** | 401471.025 | 3 | 6841.301 | 15 | 58.683 | **<0.001** |
| VegStr | 1.493 | 3 | 1.133 | 15 | 1.317 | 0.306 |
| TreeL | 878.667 | 3 | 423.108 | 15 | 2.077 | 0.146 |
| **ShrubL** | 196.742 | 3 | 39.758 | 15 | 4.948 | **0.014** |
| HerbaceousL | 173.254 | 3 | 348.006 | 15 | 0.498 | 0.689 |
| **TotalVeg** | 698.186 | 3 | 163.887 | 15 | 4.260 | **0.023** |
| NC | 1.480 | 3 | 0.730 | 15 | 2.028 | 0.153 |
| The F tests should be used only for descriptive purposes because the clusters have been chosen to maximize the differences among cases in different clusters. The observed significance levels are not corrected for this and thus cannot be interpreted as tests of the hypothesis that the cluster means are equal. | | | | | | |


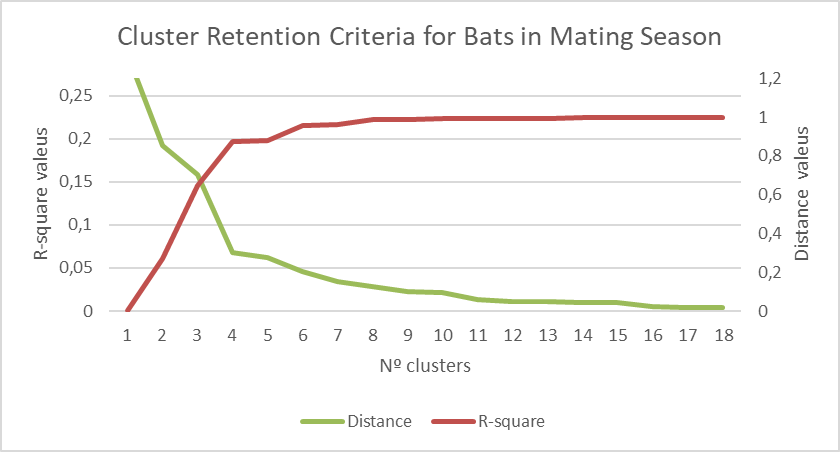


Fig. S5 – Intersection of distance between bat clusters relativized (between 0 and 1) and R- squared of each cluster, of mating analysis.

*Discriminant analysis*

*Annual analysis*

Table S5 – Standardized coefficients of variables with discriminant power of the annual discriminant analysis, the percentage of variance between the groups explained by the 3 discriminant functions extracted and the significance of the discriminant functions.

| Standardized Canonical Discriminant Function Coefficients from the annual analysis | | | |
| --- | --- | --- | --- |
|  | Function | | |
|  | 1 | 2 | 3 |
| Alt | 0.994 | 0.019 | -0.220 |
| TreeL | 0.052 | 0.777 | 1.311 |
| ShrubL | 0.157 | -0.109 | 1.418 |
| NC | -0.174 | 0.289 | -0.541 |
| *Eigenvalue* | 9.646 | 1.071 | 0.123 |
| Explained variance | 89.0 | 9.9 | 1.1 |

Table S6 - Classification results from the annual analysis, with 100,0% of original grouped cases correctly classified.

|  |  | Ecological Assemblages | Predicted Group Membership | | | | Total |
| --- | --- | --- | --- | --- | --- | --- | --- |
|  |  |  | 1.00 | 2.00 | 3.00 | 4.00 |  |
| Original | Count | Mosaic | 8 | 0 | 0 | 0 | 8 |
|  |  | Forest/edge | 0 | 11 | 0 | 0 | 11 |
|  |  | Upland | 0 | 0 | 3 | 0 | 3 |
|  |  | Aerial | 0 | 0 | 0 | 1 | 1 |
|  | % | Mosaic | 100.0 | 0.0 | 0.0 | 0.0 | 100.0 |
|  |  | Forest/edge | 0.0 | 100.0 | 0.0 | 0.0 | 100.0 |
|  |  | Upland | 0.0 | 0.0 | 100.0 | 0.0 | 100.0 |
|  |  | Aerial | 0.0 | 0.0 | 0.0 | 100.0 | 100.0 |

Table S7 - Classification Function Coefficients of the annual discriminant analysis.

| Biophysical variables | Ecological assemblage | | | |
| --- | --- | --- | --- | --- |
|  | Mosaic bats | Forest/edge bats | Upland bats | Aerial bat |
| Alt | 0.043 | 0.076 | 0.109 | 0.132 |
| TreeL | 0.781 | 0.904 | 0.813 | 0.712 |
| ShrubL | 14.958 | 16.260 | 15.302 | 18.600 |
| NC | -3.157 | -3.817 | -4.065 | -7.054 |
| (Constant) | -51.111 | -88.570 | -123.405 | -171.476 |
| Fisher's linear discriminant functions | | | | |

Table S8 - Classification functions from the annual discriminant analysis.

| Ecological assemblage | Classification function |
| --- | --- |
| Mosaic bats (MB) | MB = 0.043 Alt + 14.958 ShrubL - 3.157 NC + 0.781 TreeL - 51.111 |
| Forest/edge bats (FEB) | FEB = 0.076 Alt + 16.260 ShrubL - 3.817 NC + 0.904 TreeL - 88.570 |
| Upland bats (UB) | UB = 0.109 Alt + 15.302 ShrubL - 4.065 NC + 0.813 TreeL – 123.405 |
| Aerial bats (AB) | AB = 0.132 Alt + 18.600 ShrubL – 7.054 NC + 0.712 TreeL – 171.476 |

*Pregnancy season*

Table S9 – Standardized coefficients of variables with discriminant power of the discriminant analysis of pregnancy season, the percentage of variance between the groups explained by the 3 discriminant functions extracted and the significance of the discriminant functions.

| Standardized Canonical Discriminant Function Coefficients from the pregnancy season analysis | | |
| --- | --- | --- |
|  | Function | |
|  | 1 | 2 |
| Alt | 1.029 | -.311 |
| ShrubL | .668 | .843 |
| *Eigenvalue* | 5.631 | 0.636 |
| Explained variance | 89.9 | 10.1 |

Table S10 - Classification results from the pregnancy season analysis, with 100,0% of original grouped cases correctly classified.

|  |  | Ecological Assemblages | Predicted Group Membership | | | | Total |
| --- | --- | --- | --- | --- | --- | --- | --- |
|  |  |  | 1.00 | 2.00 | 3.00 | 4.00 |  |
| Original | Count | Mosaic | 4 | 0 | 0 | 0 | 4 |
|  |  | Forest/edge | 0 | 14 | 0 | 0 | 14 |
|  |  | Upland | 0 | 0 | 1 | 0 | 1 |
|  |  | Aerial | 0 | 0 | 0 | 1 | 1 |
|  | % | Mosaic | 100.0 | 0.0 | 0.0 | 0.0 | 100.0 |
|  |  | Forest/edge | 0.0 | 100.0 | 0.0 | 0.0 | 100.0 |
|  |  | Upland | 0.0 | 0.0 | 100.0 | 0.0 | 100.0 |
|  |  | Aerial | 0.0 | 0.0 | 0.0 | 100.0 | 100.0 |

Table S11 - Classification Function Coefficients of the discriminant analysis of the pregnancy season.

| Biophysical variables | Ecological assemblage | | | |
| --- | --- | --- | --- | --- |
|  | Mosaic bats | Forest/edge bats | Upland bats | Aerial bat |
| Alt | 0.024 | 0.043 | 0.065 | 0.071 |
| ShrubL | 5.041 | 5.951 | 10.361 | 11.474 |
| (Constant) | -15.178 | -32.484 | -81.787 | -98.134 |
| Fisher's linear discriminant functions | | | | |

Table S12 - Classification functions from the discriminant analysis of the pregnancy season.

| Ecological assemblage | Classification function |
| --- | --- |
| Mosaic bats (MB) | MB = 0.024 Alt + 5.041 ShrubL - 15.178 |
| Forest/edge bats (FEB) | FEB = 0.043 Alt + 5.951 ShrubL - 32.484 |
| Upland bats (UB) | UB = 0.065 Alt + 10.361 ShrubL - 81.787 |
| Aerial bats (AB) | AB = 0.071 Alt + 11.474 ShrubL - 98.134 |

*Nursing season*

Table S13 – Standardized coefficients of variables with discriminant power of the discriminant analysis of nursing season, the percentage of variance between the groups explained by the 3 discriminant functions extracted and the significance of the discriminant functions.

| Standardized Canonical Discriminant Function Coefficients from the nursing season analysis | | | |
| --- | --- | --- | --- |
|  | Function | |  |
|  | 1 | 2 | 3 |
| Temp | -0.202 | 0.125 | 0.055 |
| Alt | 0.969 | 0.029 | -0.217 |
| ShrubL | -0.091 | 0.750 | 0.799 |
| HerbaceousL | 0.191 | -0.504 | 0.906 |
| *Eigenvalue* | 12.490 | 0.541 | 0.045 |
| Explained variance | 95.5 | 4.1 | 0.3 |

Table S14 - Classification results from the nursing season analysis, with 100,0% of original grouped cases correctly classified.

|  |  | Ecological Assemblages | Predicted Group Membership | | | | Total |
| --- | --- | --- | --- | --- | --- | --- | --- |
|  |  |  | 1.00 | 2.00 | 3.00 | 4.00 |  |
| Original | Count | Mosaic | 4 | 0 | 0 | 0 | 4 |
|  |  | Forest/edge | 0 | 7 | 0 | 0 | 7 |
|  |  | Upland | 0 | 0 | 9 | 0 | 9 |
|  |  | Aerial | 0 | 0 | 0 | 1 | 1 |
|  | % | Mosaic | 100.0 | 0.0 | 0.0 | 0.0 | 100.0 |
|  |  | Forest/edge | 0.0 | 100.0 | 0.0 | 0.0 | 100.0 |
|  |  | Upland | 0.0 | 0.0 | 100.0 | 0.0 | 100.0 |
|  |  | Aerial | 0.0 | 0.0 | 0.0 | 100.0 | 100.0 |

Table S15 - Classification Function Coefficients of the discriminant analysis of the nursing season.

| Biophysical variables | Ecological assemblage | | | |
| --- | --- | --- | --- | --- |
|  | Mosaic bats | Forest/edge bats | Upland bats | Aerial bat |
| Temp | 8.589 | 8.229 | 7.643 | 7.510 |
| Alt | 0.034 | 0.057 | 0.094 | 0.119 |
| ShrubL | 0.678 | 0.574 | 0.500 | 0.778 |
| HerbaceousL | 0.054 | 0.071 | 0.264 | 0.127 |
| (Constant) | -103.774 | -109.085 | -137.343 | -180.374 |
| Fisher's linear discriminant functions | | | | |

Table S16 - Classification functions from the discriminant analysis of the nursing season.

| Ecological assemblage | Classification function |
| --- | --- |
| Mosaic bats (MB) | MB = 8.589 TreeL + 0.034 Alt + 0.678 ShrubL + 0.054 HerbaceousL - 103.774 |
| Forest/edge bats (FEB) | FEB = 8. 229 TreeL + 0.057 Alt + 0.574 ShrubL + 0.574 HerbaceousL - 109.085 |
| Upland bats (UB) | UB = 7.643 TreeL + 0.094 Alt + 0.500 ShrubL + 0.264 HerbaceousL - 137.343 |
| Aerial bats (AB) | AB = 7.510 TreeL + 0. 119 Alt + 0. 778 ShrubL + 0.127 HerbaceousL - 180.374 |

*Mating season*

Table S17 – Standardized coefficients of variables with discriminant power of the discriminant analysis of mating season, the percentage of variance between the groups explained by the 3 discriminant functions extracted and the significance of the discriminant functions.

| Standardized Canonical Discriminant Function Coefficients from the mating season analysis | | |
| --- | --- | --- |
|  | Function | |
|  | 1 | 2 |
| Alt | 1.052 | -0.094 |
| ShrubL | -0.250 | 1.026 |
| *Eigenvalue* | 8.445 | 1.022 |
| Explained variance | 89.2 | 10.8 |

Table S18 - Classification results from the mating season analysis, with 100,0% of original grouped cases correctly classified.

|  |  | Ecological Assemblages | Predicted Group Membership | | | | Total |
| --- | --- | --- | --- | --- | --- | --- | --- |
|  |  |  | 1.00 | 2.00 | 3.00 | 4.00 |  |
| Original | Count | Mosaic | 3 | 0 | 0 | 0 | 3 |
|  |  | Forest/edge | 0 | 12 | 0 | 0 | 12 |
|  |  | Upland | 0 | 0 | 3 | 0 | 3 |
|  |  | Aerial | 0 | 0 | 0 | 1 | 1 |
|  | % | Mosaic | 100.0 | 0.0 | 0.0 | 0.0 | 100.0 |
|  |  | Forest/edge | 0,0 | 100,0 | 0,0 | 0,0 | 100,0 |
|  |  | Upland | 0,0 | 0,0 | 100,0 | 0,0 | 100,0 |
|  |  | Aerial | 0,0 | 0,0 | 0,0 | 100,0 | 100,0 |

Table S19 - Classification Function Coefficients of the discriminant analysis of the mating season.

| Biophysical variables | Ecological assemblage | | | |
| --- | --- | --- | --- | --- |
|  | Mosaic bats | Forest/edge bats | Upland bats | Aerial bat |
| Alt | 0.079 | 0.122 | 0.166 | 0.178 |
| ShrubL | -0.053 | -0.360 | -0.634 | 0.007 |
| (Constant) | -30.961 | -66.025 | -122.607 | -156.408 |
| Fisher's linear discriminant functions | | | | |

Table S20 - Classification functions from the discriminant analysis of the mating season.

| Ecological assemblage | Classification function |
| --- | --- |
| Mosaic bats (MB) | MB = 0.079 Alt - 0.053 ShrubL - 30.961 |
| Forest/edge bats (FEB) | FEB = 0.122 Alt - 0.360 ShrubL - 66.025 |
| Upland bats (UB) | UB = 0.166 Alt – 0.634 ShrubL – 122.607 |
| Aerial bats (AB) | AB = 0.178 Alt + 0.007 ShrubL – 156.408 |

*Bat species vulnerability*


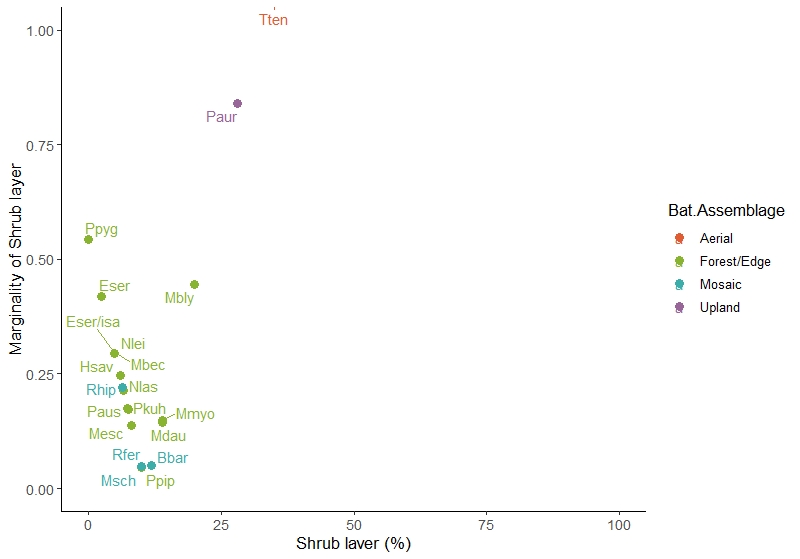

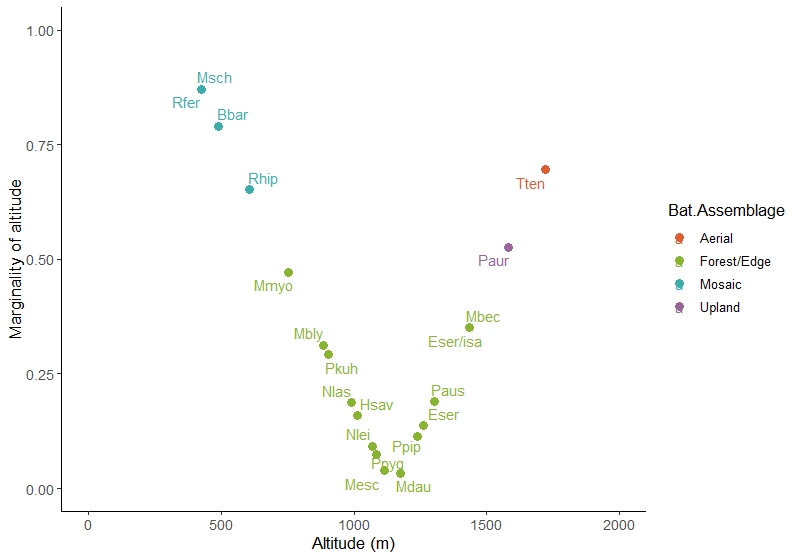


**Pregnancy**

Fig.S6 - Relationship between marginality and weighted average of each significant variables, extracted from the discriminant analysis of the pregnancy season (Altitude and Shrub layer) (Table S19).


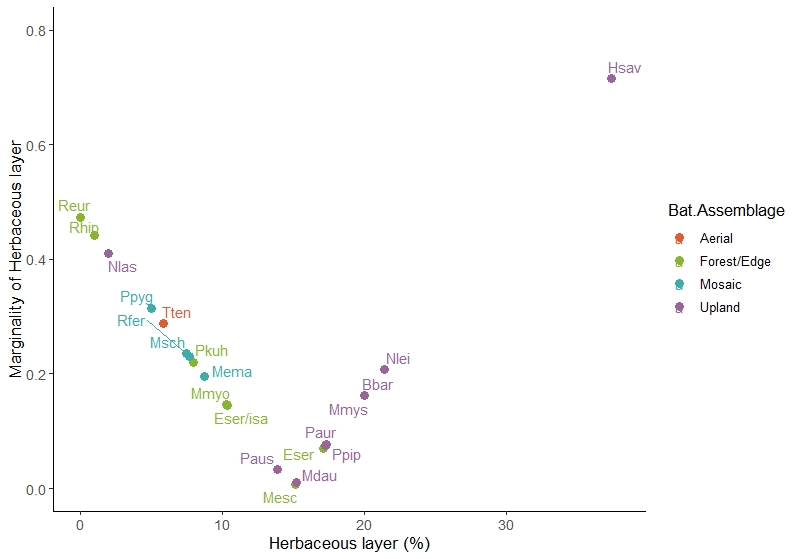

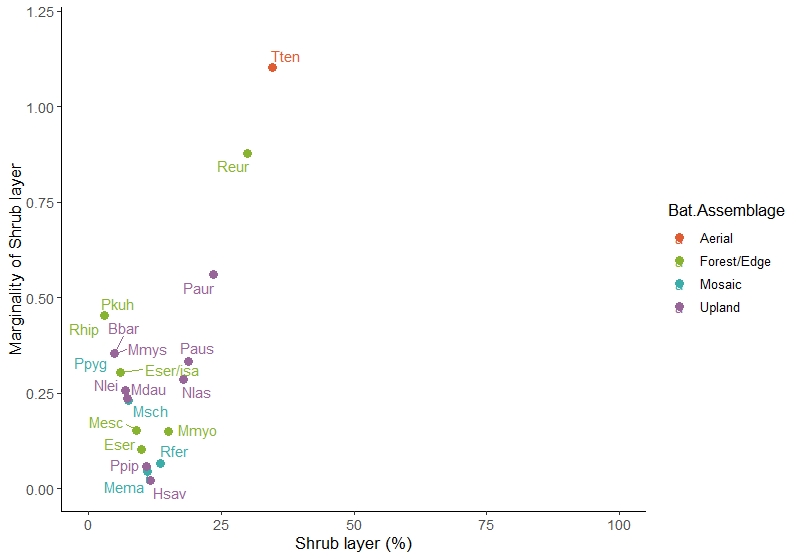

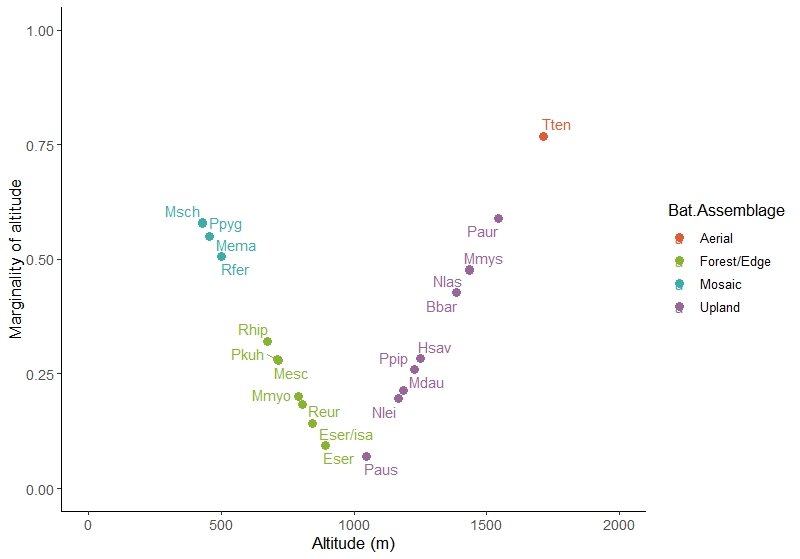

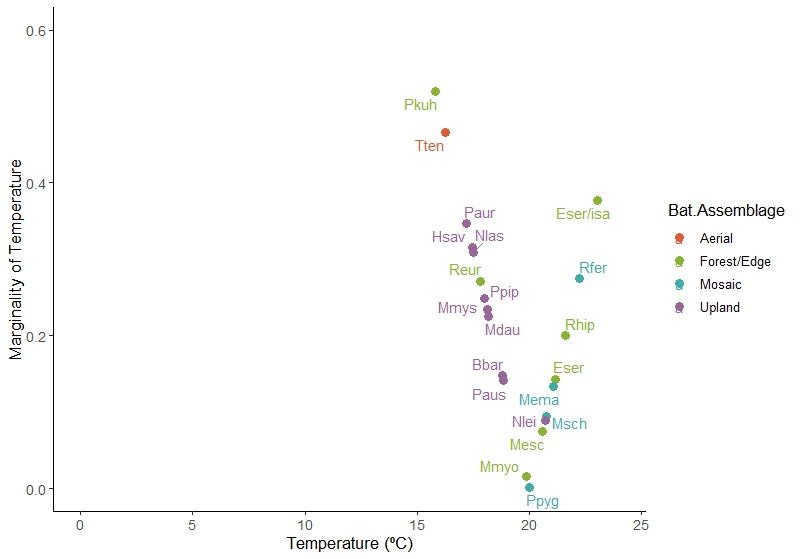


**Nursing**

Fig.S7 - Relationship between marginality and weighted average of each significant variables, extracted from the discriminant analysis of the nursing season (Altitude, Shrub layer, Herbaceous layer and Temperature) (Table S13).

**Mating**


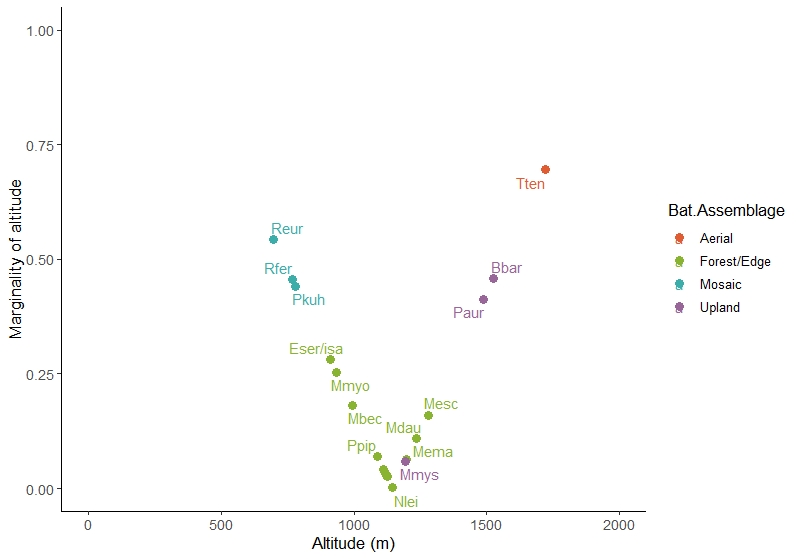

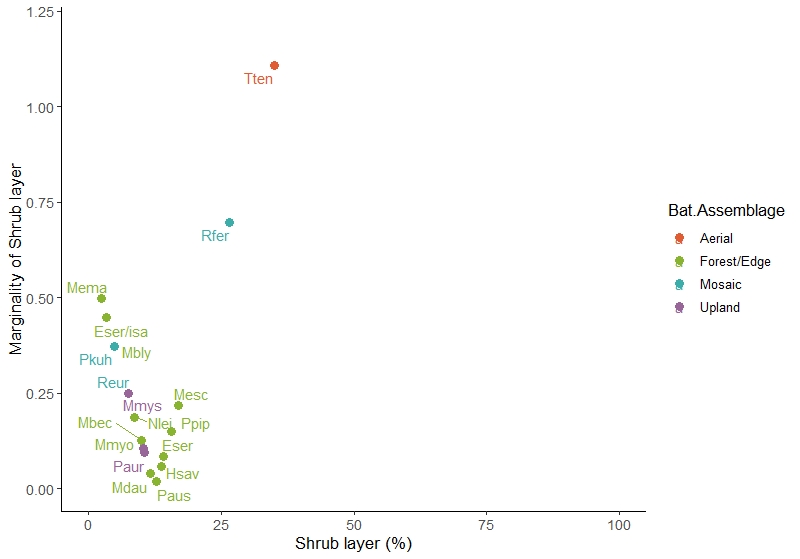


Fig.S8 - Relationship between marginality and weighted average of each significant variables, extracted from the discriminant analysis of the mating season (Altitude and Shrub layer) (Table S17).

Table S21 - National conservation status of bat species analyzed in the case study.

| **Species** | **Acronym** | **National conservation status** |
| --- | --- | --- |
| *Myotis mystacinus* | Mmys | VU |
| *Myotis bechsteinii* | Mbec | DD |
| *Nyctalus lasiopterus* | Nlas | DD |
| *Nyctalus leisleri* | Nlei | LC |
| *Barbastella barbastellus* | Bbar | LC |
| *Myotis daubentonii* | Mdau | LC |
| *Rhinolophus euryale* | Reur | EN |
| *Eptesicus serotinus* | Eser | LC |
| *Pipistrellus kuhlii* | Pkul | LC |
| *Myotis blythii* | Mbly | CR |
| *Pipistrellus pipistrellus* | Ppip | LC |
| *Plecotus austriacus* | Paus | NT |
| *Myotis escalerai* | Mesc | VU |
| *Hypsugo savii* | Hsav | LC |
| *Pipistrellus pygmaeus* | Ppyg | LC |
| *Myotis myotis* | Mmyo | VU |
| *Rhinolophus hipposideros* | Rhip | LC |
| *Miniopterus schreibersii* | Msch | NT |
| *Tadarida teniotis* | Tten | LC |
| *Plecotus auritus* | Paur | DD |
| *Rhinolophus ferrumequinum* | Rfer | LC |
| *Myotis emarginatus* | Mema | EN |

Table S22 - Weighted average and marginality of each species of the environment variables that major influence bat species to assess their vulnerability by graphical correlation in the annual analysis.

| Species | Weighted average for altitude | Marginality for altitude | Weighted average for tree layer | Marginality for tree layer | Weighted average for shrubs layer | Marginality for shrubs layer | Weighted average for night cooling | Marginality for night cooling | Bat Assemblage |
| --- | --- | --- | --- | --- | --- | --- | --- | --- | --- |
| Rfer | 561.05 | 0.65 | 23.86 | 0.36 | 16.78 | 0.20 | 2.06 | 0.02 | Mosaic |
| Msch | 427.33 | 0.81 | 36.67 | 0.15 | 10.00 | 0.09 | 1.47 | 0.11 | Mosaic |
| Mbec | 1216.00 | 0.12 | 75.00 | 0.45 | 5.00 | 0.31 | 2.35 | 0.10 | Forest/Edges |
| Mesc | 1030.37 | 0.10 | 45.94 | 0.01 | 10.91 | 0.05 | 2.84 | 0.20 | Forest/Edges |
| Mmyo | 809.25 | 0.36 | 36.70 | 0.15 | 14.30 | 0.09 | 1.04 | 0.21 | Forest/Edges |
| Eser | 991.81 | 0.14 | 44.42 | 0.03 | 10.81 | 0.06 | 1.76 | 0.05 | Forest/Edges |
| Eser/isa | 966.33 | 0.17 | 68.17 | 0.35 | 5.00 | 0.31 | 3.08 | 0.25 | Forest/Edges |
| Mbly | 965.33 | 0.18 | 36.67 | 0.15 | 15.00 | 0.13 | 1.10 | 0.20 | Forest/Edges |
| Paur | 1535.09 | 0.49 | 23.77 | 0.36 | 20.64 | 0.37 | 1.26 | 0.16 | Upland |
| Mmys | 1436.00 | 0.38 | 70.00 | 0.38 | 5.00 | 0.31 | 2.82 | 0.20 | Upland |
| Tten | 1721.00 | 0.71 | 0.00 | 0.74 | 35.00 | 0.99 | 1.58 | 0.10 | Aerial |
| Mema | 563.29 | 0.65 | 28.93 | 0.28 | 10.36 | 0.08 | 0.71 | 0.28 | Mosaic and Forest/Edges |
| Pkuh | 826.00 | 0.34 | 36.75 | 0.15 | 5.75 | 0.28 | 2.00 | 0.01 | Mosaic and Forest/Edges |
| Reur | 759.21 | 0.42 | 46.43 | 0.00 | 20.36 | 0.36 | 3.50 | 0.34 | Mosaic and Forest/Edges |
| Rhip | 620.20 | 0.58 | 45.80 | 0.01 | 5.80 | 0.27 | 0.84 | 0.25 | Mosaic and Forest/Edges |
| Ppyg | 758.00 | 0.42 | 35.00 | 0.18 | 5.00 | 0.31 | 1.40 | 0.13 | Mosaic and Forest/Edges |
| Ppip | 1174.26 | 0.07 | 48.37 | 0.03 | 12.82 | 0.03 | 1.74 | 0.05 | Forest/Edges and Upland |
| Hsav | 1121.70 | 0.01 | 43.32 | 0.05 | 10.91 | 0.05 | 1.46 | 0.11 | Forest/Edges and Upland |
| Nlei | 1141.01 | 0.03 | 71.24 | 0.39 | 7.34 | 0.21 | 1.74 | 0.05 | Forest/Edges and Upland |
| Nlas | 1101.50 | 0.02 | 67.50 | 0.34 | 9.50 | 0.11 | 2.03 | 0.01 | Forest/Edges and Upland |
| Paus | 1158.69 | 0.05 | 37.40 | 0.14 | 13.58 | 0.06 | 2.41 | 0.10 | Forest/Edges and Upland |
| Mdau | 1199.75 | 0.10 | 54.55 | 0.13 | 11.00 | 0.05 | 2.62 | 0.15 | Forest/Edges and Upland |
| Bbar | 1466.77 | 0.41 | 52.97 | 0.11 | 9.10 | 0.13 | 1.82 | 0.03 | Mosaic and Upland |

.


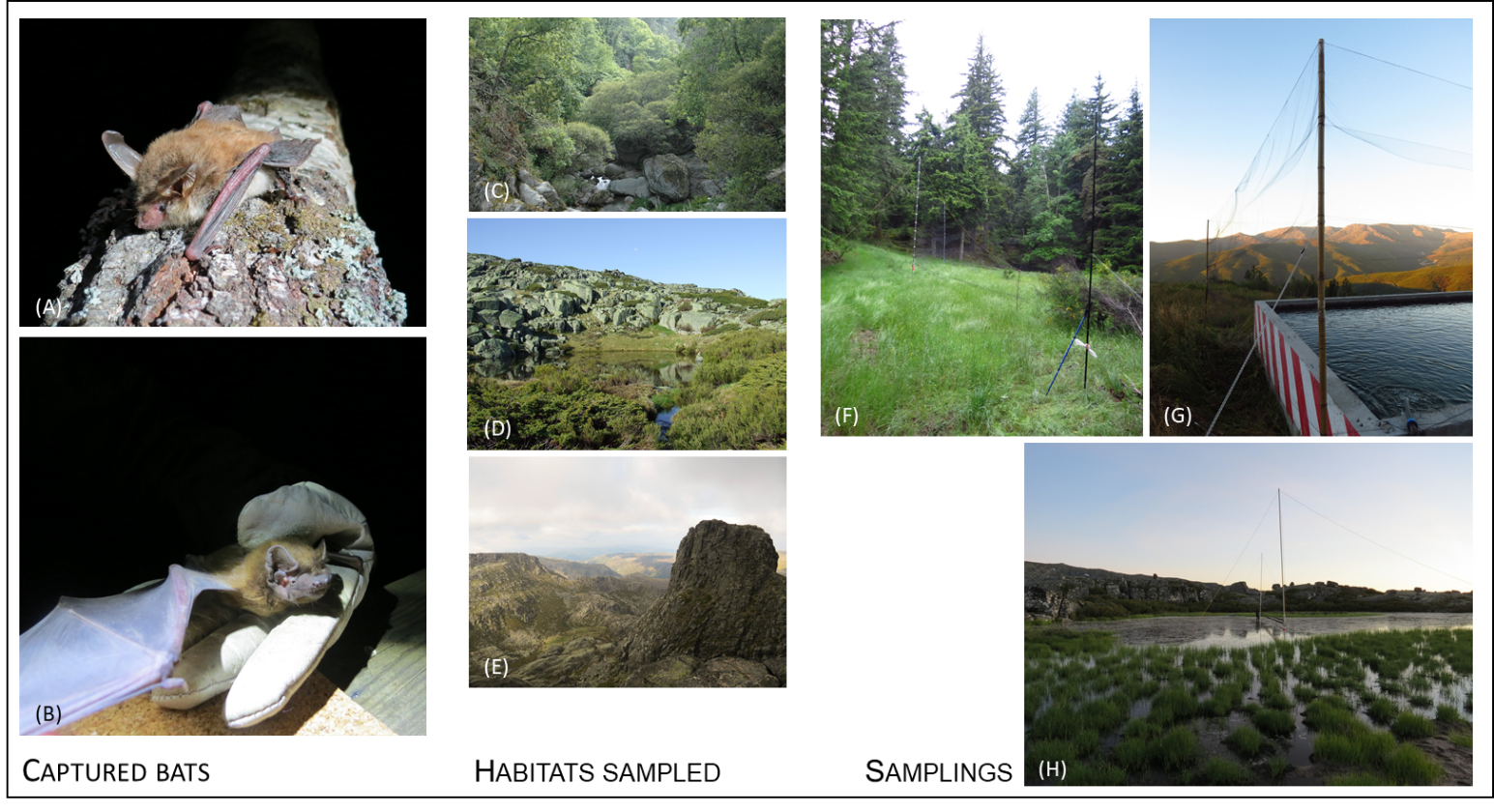
Fig. S9 - Pictures of some captured bats (A – *M. bechsteinii* and B – *N. lasiopterus*), sampled habitats (C - mountain forest and river, D – mountain shrubs and E - mountain rupicolous habitat) and some samplings performed during the fieldwork (F- sampling in a mountain forest, G - sampling in a firefighting water tank and H – sampling in a natural lake).

Table S23 – Dates of visitation of each sampling site, type and number of visitations.

**References**

Maroco, J. 2010. 'Análise estatística com utilização do SPSS Lisboa: Sílabo p. 822', ISBN: 978-972-618-452-2.
