# Supplementary material for: Changing with the times: Seasonal environmental gradients unveil dynamic bat assemblages and vulnerability
Source: Ecol Evol. 2023 Jul 17;13(7):e10246. doi: 10.1002/ece3.10246 (PMC10352094; doi:10.1002/ece3.10246)
Supplement: Supplementary file 2 — Data S1 [file ECE3-13-e10246-s002.docx]

.      Supplementary Information dataset citation and caption:

The dataset was only submitted for review by the reviewers. As described in the Data Accessibility Statement, "the authors compromise to publish the dataset analyzed in this manuscript in a publicly accessible repository after its acceptance." This was done in the Dryad platform as the following link shows:
[https://datadryad.org/stash/dataset/doi:10.5061/dryad.d2547d84w](https://urldefense.com/v3/__https:/datadryad.org/stash/dataset/doi:10.5061/dryad.d2547d84w__;!!N11eV2iwtfs!ockxQOPlAAktYnTLIWeOzw9ZGoftFMyfgSKyQsMEfw84tWLhXul8KHhsLuIWVGmXBwU5qeyQhmlD06pgVTCkyIw4$)

citation: figure 1 caption.
